# Multiphysics and multiscale modeling of microthrombosis in COVID-19

**DOI:** 10.1371/journal.pcbi.1009892

**Published:** 2022-03-07

**Authors:** He Li, Yixiang Deng, Zhen Li, Ander Dorken Gallastegi, Christos S. Mantzoros, Galit H. Frydman, George E. Karniadakis

**Affiliations:** 1 School of Engineering, Brown University, Providence, Rhode Island, United States of America; 2 Department of Mechanical Engineering, Clemson University, Clemson, South Carolina, United States of America; 3 Department of Surgery, Department of Emergency Medicine and Department of Medicine, Massachusetts General Hospital, Boston, Massachusetts, United States of America; 4 Department of Medicine, Boston VA Healthcare system and Beth Israel Deaconess Medical Center, Harvard Medical School, Boston, Massachusetts, United States of America; 5 Division of Trauma, Emergency Surgery and Surgical Critical Care at the Massachusetts General Hospital, Boston, Massachusetts, United States of America; 6 Center for Biomedical Engineering at the Massachusetts Institute of Technology, Cambridge, Massachusetts, United States of America; 7 Division of Applied Mathematics, Brown University, Providence, Rhode Island, United States of America; Stanford University, UNITED STATES

## Abstract

Emerging clinical evidence suggests that thrombosis in the microvasculature of patients with Coronavirus disease 2019 (COVID-19) plays an essential role in dictating the disease progression. Because of the infectious nature of SARS-CoV-2, patients’ fresh blood samples are limited to access for *in vitro* experimental investigations. Herein, we employ a novel multiscale and multiphysics computational framework to perform predictive modeling of the pathological thrombus formation in the microvasculature using data from patients with COVID-19. This framework seamlessly integrates the key components in the process of blood clotting, including hemodynamics, transport of coagulation factors and coagulation kinetics, blood cell mechanics and adhesive dynamics, and thus allows us to quantify the contributions of many prothrombotic factors reported in the literature, such as stasis, the derangement in blood coagulation factor levels and activities, inflammatory responses of endothelial cells and leukocytes to the microthrombus formation in COVID-19. Our simulation results show that among the coagulation factors considered, antithrombin and factor V play more prominent roles in promoting thrombosis. Our simulations also suggest that recruitment of WBCs to the endothelial cells exacerbates thrombogenesis and contributes to the blockage of the blood flow. Additionally, we show that the recent identification of flowing blood cell clusters could be a result of detachment of WBCs from thrombogenic sites, which may serve as a nidus for new clot formation. These findings point to potential targets that should be further evaluated, and prioritized in the anti-thrombotic treatment of patients with COVID-19. Altogether, our computational framework provides a powerful tool for quantitative understanding of the mechanism of pathological thrombus formation and offers insights into new therapeutic approaches for treating COVID-19 associated thrombosis.

## Author summary

Emerging clinical evidence suggests that thrombosis in the microvasculature of patients with Coronavirus disease 2019 (COVID-19) plays an essential role in dictating the disease progression. We employ a novel multiphysics and multiscale computational framework to investigate the underlying mechanism of the pathological formation of microthrombi and circulating cell clusters in COVID-19. We quantify the contributions of many prothrombotic factors reported in the literature, such as stasis, the derangement in blood coagulation factor levels and activities, inflammatory responses of endothelial cells and leukocytes to the microthrombus formation in COVID-19, through which we identify the potential targets that should be further evaluated, and prioritized in the anti-thrombotic treatment of patients with COVID-19.

## Introduction

Coronavirus disease 2019 (COVID-19), caused by infection with severe acute respiratory syndrome coronavirus 2 (SARS-CoV-2), continues to be a major cause of mortality throughout the world. Emerging clinical data suggests that patients with COVID-19 are prone to develop venous thrombosis, which may progress to pulmonary embolism [1], as well as arterial thrombosis that is likely responsible for the increased risk of stroke or myocardial infarction [2]. In addition to macrovascular thrombosis, a strong association of COVID-19 with microvascular thrombosis was evidenced by multiple studies based on postmortem examinations of the lungs [3–5]. These studies showed the prevalence of microthrombi in the small pulmonary vasculature in 80% to 100% cases of people who died from COVID-19, suggesting that severe COVID-19 is a microvascular disease [5]. Further autopsy findings of microthrombi in other organ systems, including the heart, kidneys and liver, indicate that microthrombosis may contribute to multi-organ damage and failure in the severe cases of COVID-19 [6–8].

Both the macrovascular and microvascular thrombotic complications in COVID-19 indicate a strong interplay between the SARS-CoV-2 and activation of coagulation [9, 10]. All three components of Virchow’s triad, namely stasis, endothelial injury, and hypercoagulable state, are likely to contribute to the increased thrombotic risk of patients with COVID-19 [9]. Stasis may result from the reduced mobilization of patients who are critically ill as well as from physical obstruction (partial or total) of a blood vessel by clots or blood cell clusters. Endothelial injury or death as a consequence of the direct viral invasion results in exposure of various extracellular matrix proteins that can activate platelets and initiate the coagulation cascade [11]. In addition, viral infection can trigger inflammatory responses to stimulate endothelial cells to upregulate the expression of tissue factor (TF), a prime activator of the coagulation cascade [12]. A hypercoagulable state of patients with COVID-19 was manifested as the derangement in the level of zymogens, cofactors, and endogenous anticoagulants, such as increased levels of factor V (FV), factor VIII (FVIII), factor X (FX), fibrinogen (FI), protein C (PC) and decreased amount of antithrombin (AT) (see Table 1).

**Table 1:** Aberrant levels of coagulation factors in COVID-19.

Factors	Values in patients with COVID-19	Reference range	References
	Mean (min-max or std)	Mean (min-max or std)	
FI (mg/dL)	680 (234–1344)	258 (165–350)	[10]
	760 (170)	N/A	[18]
	517 (148)	297 (78)	[19]
	660 (190)	300 (150–450)	[20]
	810 (640–945)	<400	[21]
AT (U/dL)	74 (45–120)	102 (82–122)	[10]
	72.2 (23.4) (Non-survivors)	N/A	[22]
	94.6(19.5) (Survivors)	N/A	[22]
	70.6 (23.7)	105 (70–140)	[20]
	83 (66–94)	100 (80–120)	[21]
FVIII (U/dL)	199 (100–369)	102.5 (52–153)	[23]
	297 (223–470)	99 (51–147)	[10]
	341 (258–416)	105 (60–150)	[24]
	297 (214–364)	90 (60–120)	[21]
FV (U/dL)	150	105 (60–150)	[25]
	136 (115–150)	>70	[24]
	153 (122–172)	95 (70–120)	[21]
FX (U/dL)	115 (101–119)	95 (70–120)	[21]
PC (U/dL)	122 (75–177)	113 (60–165)	[10]

In the last few decades, the traditional notion that hemostatic system is solely regulated by the coagulation cascade coupled with platelet activation has been challenged by increased evidence showing that the immune system also intertwines with blood coagulation and thrombus formation [13]. The roles of immune cells like platelets and white blood cells (WBCs) in immunothrombosis have been further witnessed in COVID-19 pandemic [9]. The injured endothelial cells, as a result of viral invasion, not only activate and subsequently bind to platelets, but also attract WBCs like neutrophils and monocytes by expressing P-selectin, a transmembrane protein that binds with its major ligand P-selectin glycoprotein ligand-1 (PSGL-1) on the plasma membranes of WBCs, contributing to the firm adhesion of WBCs to endothelial cells and their subsequent activation [14, 15]. Activated WBCs can expose procoagulant factors that initiate blood coagulation and release cytokines that modulate the expression of procoagulants and adhesive molecules on endothelial cells [13]. The cytokine released by immune cells and injured endothelial cells contribute to the cytokine storm [16], a severe immune reaction which is thought to cause extrapulmonary thrombotic complications in COVID-19 [17].

In this work, we employ a multiphysics and multiscale computational framework to simulate the formation of microthrombi and circulating cell clusters (CCCs) triggered by multiple prothrombotic factors reported in the literature, such as increased level of inflammation, stasis, the derangement in blood coagulation factor levels and activities, as well as the adhesion and activation of WBCs, and quantify the contributions of these factors to the extent of microthrombosis in COVID-19. This novel framework considers the key components involved in the process of thrombus formation, including hemodynamics, transport of coagulation factors and coagulation kinetics, blood cell mechanics, platelet and WBC adhesive dynamics, which allows us to dissect the complex multiphysics processes involved in the microthrombosis and identify the key factors that regulate these processes, thereby providing insights into more effective and tailored antithrombotic strategies to treat patients with COVID-19.

## Models and methods

A number of computational models for red blood cells (RBCs) and platelets have been developed in the past two decades to explore the biological processes associated with blood disorders and thrombosis, see recent reviews [26–31]. The protein-level RBC and platelet models, such as [32–35], are capable of investigating the pathological alterations of the biomechanics of diseased RBCs resulting from either protein defects or virus invasion [36–41]. However, these models cannot be employed to simulate blood cell suspensions or blood flow due to the high computational cost. On the other hand, highly efficient cellular-level RBC and platelet models developed based on [42] have been widely used to study multiscale biological phenomenons from single RBC mechanics [38, 43] to blood flow dynamics [44, 45], but they are lack of the biochemical component to describe the coagulation-induced platelet activation. In this work, we implement a multiphysics and multiscale computational framework based on our previous work [46] to investigate the impacts of multiple prothrombotic factors, such as stasis, inflammatory responses of endothelial cells, aberrant levels of coagulation factors, involvement of WBCs, on microthrombosis in COVID-19. Distinct from numerous computational models for simulating blood coagulation, platelet activation and aggregation as well as thrombus formation [47–56], which are summarized in recent reviews [31, 57, 58], the employed framework integrates four main modules involving in the coagulation-induced thrombosis, namely 1) the hemodynamics, 2) blood cell mechanics, 3) platelet and WBC adhesive dynamics, 4) transport of coagulation factors and coagulation kinetics, on a single computational framework (LAMMPS: Molecular Dynamics Simulator) and these modules are introduced in the following sections. In the current work, we focus on investigating the impacts of 1) aberrant levels of coagulation factors measured from the patients with COVID-19 as listed in Table 1; 2) hemodynamics; 3) involvement of WBCs on the initiation and development of microthrombosis in COVID-19.

### Hemodynamics and blood cell mechanics

Blood plasma in the simulation is modeled using dissipative particle dynamics (DPD) method following our previous work [59]. Detailed information of the DPD method can be found in S1 Text and the model parameters are listed in Table A in S1 Text.

**Fig 1:**
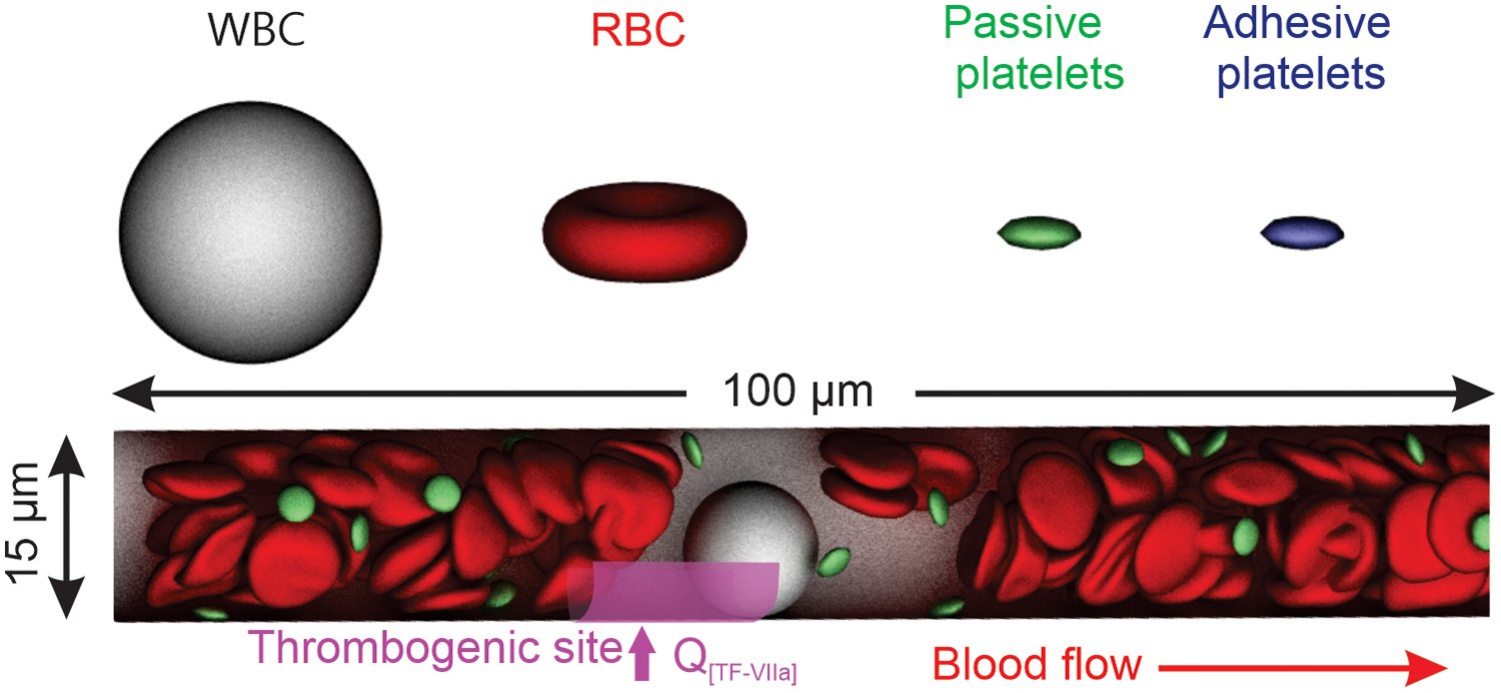
Schematics of the simulation setup. Top row: particle-based models for WBCs, RBCs and platelets. Second row: A 3D circular microchannel with a diameter of 15 *μ*m and a length of 100 *μ*m is filled with RBCs, platelets, one WBC and solvent particles which carry 23 factors. Solvent particles and RBCs close to the WBC are omitted in visualization for clarity. One thrombogenic site (highlighted in magenta) with a length of 30 *μ*m is assigned on the vessel wall. Mass fluxes of four factors (listed in Table C in S3 Text) depending on the level of [TF-VIIa] are injected into the channel from the thrombogenic site to initiate and drive the coagulation cascade. Blood flows from left to right.

We employ a particle-based model introduced in the work of Fedosov et al. [59] to represent RBCs and use its extension for platelets and WBCs, as shown in Fig 1(top row). The membrane of RBCs, platelets and WBCs is constructed by a 2D triangulated network with *N*_*v*_ vertices (DPD particles). The vertices are connected by *N*_*s*_ elastic bonds to impose proper membrane elasticity. A bending potential is imposed to control the bending stiffness of the cell membrane. In addition, the area and volume constraints are imposed to mimic the area-preserving lipid bilayer and the incompressible interior fluid. The detailed formulation of potentials implemented in the blood cell models is introduced in S1 Text and the corresponding model parameters are listed in Table B in S1 Text.

### Platelet and WBC adhesive dynamics

The platelet and WBC adhesion models describe the adhesive dynamics of receptors on the platelet or WBC membrane binding to their ligands. In this work, we employ the adhesive dynamics model that utilizes the Monte Carlo method to determine each bond formation/breakage event based on specific receptor-ligand binding kinetics [60]. We estimate the probabilities of bond formation *P*_*f*_ and bond breakage *P*_*r*_ using the following equations

$$P_{f}=1-\text{exp}(-k_{f}\Delta t),$$ (1a)

$$P_{r}=1-\text{exp}(-k_{r}\Delta t),$$ (1b)

where *k*_*f*_ and *k*_*r*_ are the rates of formation and breakage, respectively. To describe the adhesive dynamics between platelets as well as between platelets and vessel wall, we employ the catch bond formula proposed by Mody and King [60] where the force-dependent *k*_*f*_ and *k*_*r*_ are evaluated by

$$\begin{array}{c} \begin{array}{cc} k_{f} & =k_{f}^{0}\text{exp}\left(|F_{b}|\frac{\left(\sigma_{f}-0.5|l-l_{0}|\right)}{k_{B}T}\right),\\ k_{r} & =k_{r}^{0}\text{exp}\left(\frac{\sigma_{r}|F_{b}|}{k_{B}T}\right), \end{array} \end{array}$$ (2)

where $k_{f}^{0}$ and $k_{r}^{0}$ are the intrinsic bond formation and breakage rates, respectively; *l* and *l*_0_ are bond length and equilibrium bond length, respectively; *F*_*b*_ is the bond force; *σ*_*f*_ and *σ*_*r*_ are the corresponding reactive compliance. The values of these model parameters are listed in Table A in S2 Text. As for the adhesive dynamics between WBCs and platelets as well as between WBCs and vessel wall, we employ the formulation developed by Dembo et al. [61], which has been widely used to simulate the rolling and adhesion of WBCs to the endothelial cells [62, 63]. The forward and reverse rates for the receptor-ligand bond are calculated as

$$\begin{array}{c} \begin{array}{cc} k_{f} & =k_{f}^{0}\text{exp}\left(-\frac{\sigma_{ts}{\left(l-l_{0}\right)}^{2}}{2k_{B}T}\right),\\ k_{r} & =k_{r}^{0}\text{exp}\left(\frac{\left(\sigma_{b}-\sigma_{ts}\right){\left(l-l_{0}\right)}^{2}}{2k_{B}T}\right), \end{array} \end{array}$$ (3)

where *σ*_*b*_ is the spring constant and *σ*_*ts*_ is the spring constant in the transition states. The values of these model parameters are listed in Table B in S1 Text. More detailed information of the platelet and WBC adhesive dynamics models can be found in S1 Text.

### Transport of coagulation factors and coagulation kinetics

Transport of coagulation factors involved in the cascade is modeled by transport Dissipative Particle Dynamics (tDPD) [64], an extension of the classical DPD framework to consider the chemical concentration fields. We implement the mathematical model of Anand et al. [65] to describe the coagulation kinetics and this model contains a set of 21 coupled diffusion-reaction equations to describe the evolution of 23 biological factors involved in both intrinsic and extrinsic pathways of blood coagulation (see Tables A and B in S3 Text). Two key products of this coagulation cascade are FIIa, which mediates platelet activation and coagulation cascade, and FIa, an essential component of the blood clots. Detailed information of the tDPD model and the model of Anand et al. [65] can be found in S3 Text.

### Problem setup

In this work, we focus on simulating the initiation of microthrombosis, a prominent clinical feature in COVID-19. As illustrated in Fig 1(second row), we set up a 100 *μm* long circular microchannel with a diameter of 15 *μm*. A periodic boundary condition is imposed along the flow direction. This microchannel is filled with RBCs, platelets and solvent particles which carry 23 coagulation factors. The initial concentrations of these factors are listed in Table A in S3 Text. The hematocrit of the blood is selected to be ∼40%. All platelets are initially placed close to the channel wall to accelerate margination and the adhesion process. Passive platelets are occasionally inserted into the system from the inlet of the vessel to compensate the reduced number of flowing platelets due to their adhesion to the thrombogenic site. No-slip condition is implemented on the channel wall by treating the wall as pseudo-planes where particles within one cut-off distance from the wall are reflected in a bounce-forward scheme [66].

We start the simulation by first allowing the blood flow to reach equilibrium without initiating the coagulation cascade. A variety of pressure gradients are applied to each DPD particle to simulate blood flow with mean velocities varying from 0.165 mm/s to 1.76 mm/s, covering both the stasis and physiologically-relevant flow conditions in the microvasculature [67–69]. Once the blood flow reaches equilibrium, we impose boundary fluxes of four factors that depend on the level of TF-VIIa complex at the thrombogenic site (Fig 1) to mimic the initiation of the coagulation cascade by TF expressed on the endothelial cells in response to inflammation. Detailed information of influx boundary conditions at the thrombogenic site are listed in Table C in S3 Text. Following the work of Papadopoulos et al. [70], we set a threshold value of 1 nM for FIIa-mediated platelet activation, i.e., platelets instantaneously become activated when FIIa concentration exceeds 1 nM. Activated platelets are adhesive and able to form bonds with the particles representing vWF ligands at the thrombogenic site as well as with other activated platelets and WBCs. The activated platelets also release adenosine diphosphate (ADP) that contributes to the activation of passive platelets flowing in the blood. More details of platelet activation mechanism can be found in S3 Text.

## Results

### Sensitivity analysis of the aberrant levels of coagulation factors in COVID-19

**Fig 2:**
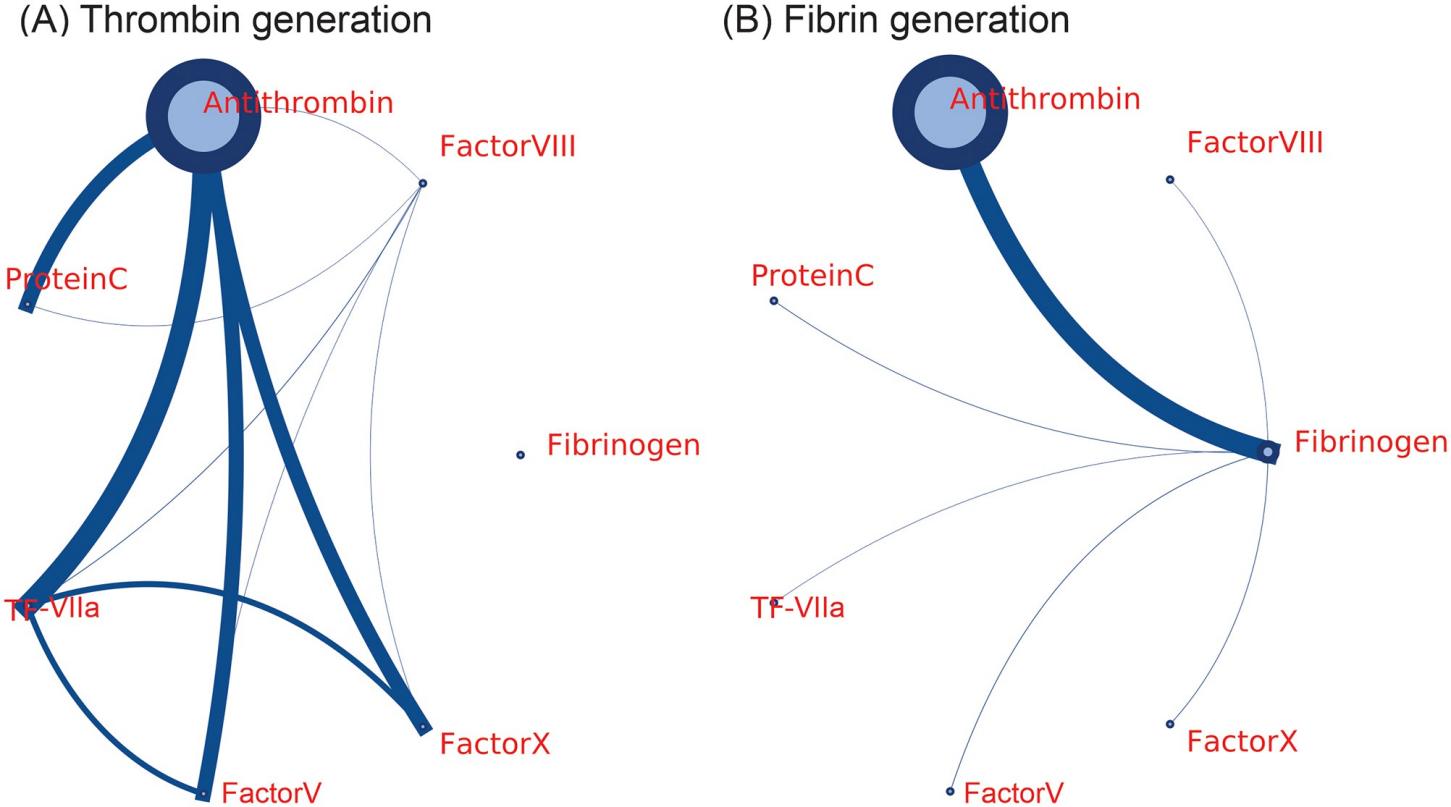
Global sensitivity analysis of how aberrant levels of coagulation factors in COVID-19 affect the generation of FIIa (A) and FIa (B). Circles in the figure signify the sensitivity of (A) thrombin and (B) fibrin to a single varying factor and the diameters of the circles correspond to values of the sensitivity for the examined factors. The lines that connect two factors designate the sensitivity of (A) thrombin and (B) fibrin to the two connected factors when they are varied synchronously. The thickness of the lines reflect the values of the sensitivity.

Patients who are hospitalized with severe COVID-19 infections showed increased levels of FI, FVIII, FV, FX, PC and decreased amount of AT compared to the normal subjects (see Table 1). In this section, we employ global sensitivity analysis to investigate how these altered coagulation factors in COVID-19 impact the generation of thrombin (coagulation factor IIa [FIIa]) and fibrin (coagulation factor Ia [FIa]), two essential elements in blood clotting. To perform sensitivity analysis, we employ the analysis of variance functional decomposition [71], where we vary seven coagulation factors simultaneously, namely FI, FV, FVIII, FX, AT, PC and TF-coagulation factor VIIa (FVIIa) complex (TF-VIIa). Guided by the values listed in Table 1, we select the ranges of variation for the first six factors to be between the extreme values of these factors measured from the patients with COVID-19 and the mean reference values. TF-VIIa, representing the level of inflammatory responses of endothelial cells in our simulation, is varied between 0.001–0.2 nM, following the work of Anand et al. [65]. To illustrate the results of the sensitivity analysis, we place the examined coagulation factors in a ring, as shown in Fig 2A and 2B. The sensitivity of a single factor is plotted as a circle, whose diameter reflects the sensitivity of the FIIa/FIa generation to that factor. The connecting lines indicate the interaction of two factors, which describe how FIIa/FIa generation changes when these two factors are varied synchronously. The thickness of a line denotes the sensitivity of the pair.

First, we investigate the sensitivity of FIIa generation to the seven considered coagulation factors. Our results in Fig 2A indicate that FIIa generation is predominately dictated by the level of AT when each factor varies separately. In the case of two factors varying synchronously, FIIa generation is most sensitive to the pair of AT-TF-VIIa. Fig 2B shows that FIa generation is also most sensitive to the level of AT, followed by concentration of FI for single factor analysis, while AT-FI pair appears to be most essential in determining the generation of FIa. These results suggest that the effect of the decreased level of AT on promoting coagulation-initiated thrombosis overwhelms the inhibiting impact of the increased level of PC in COVID-19. This finding is consistent with prior experimental studies which demonstrated that AT deficiency has a more profound effect on accelerating thrombus growth than PC deficiency [72]. These results also provide a therapeutic rationale for using low molecular weight heparin (LMWH) and unfractionated heparin (UFH), a group of anticoagulants that inhibit coagulation by activating AT to suppress the conversion from FX and FII to FXa and FIIa, to treat thrombosis in COVID-19 [73]. Preliminary results have shown that LMWH treatment reduced the mortality in patients with COVID-19 with an elevated D-dimer or elevated sepsis-induced coagulopathy score [74].

### Elevated level of inflammatory responses of endothelial cells and reduced blood flow rates promote thrombus formation

In the last section, we performed sensitivity analysis of aberrant coagulation factors in COVID-19 on FIIa/FIa generation without considering the impact of the blood flow. Flowing blood can bring in inactive factors, such as prothrombin (FII) and FV, to coagulation reaction and remove active factors, such as FIIa. Thus, there is a competition between the rate of coagulation reactions and the strength of the advection of coagulation factors, leading to a threshold response of thrombus development to blood flow shear rate [75]. In this section, we simulate thrombus formation in a microvessel with a diameter of 15 *μm*, corresponding to the size of postcapillary venules. The mean blood flow velocities in the microvessel are varied from 0.165 to 1.75 mm/s to quantify the impact of hemodynamics on the thrombus development. These examined blood flow velocities result in wall shear rates in a range of ∼90–950 *s*^−1^, which covers both stasis and physiologically-relevant flow conditions in microvasculature [67, 69].

**Fig 3:**
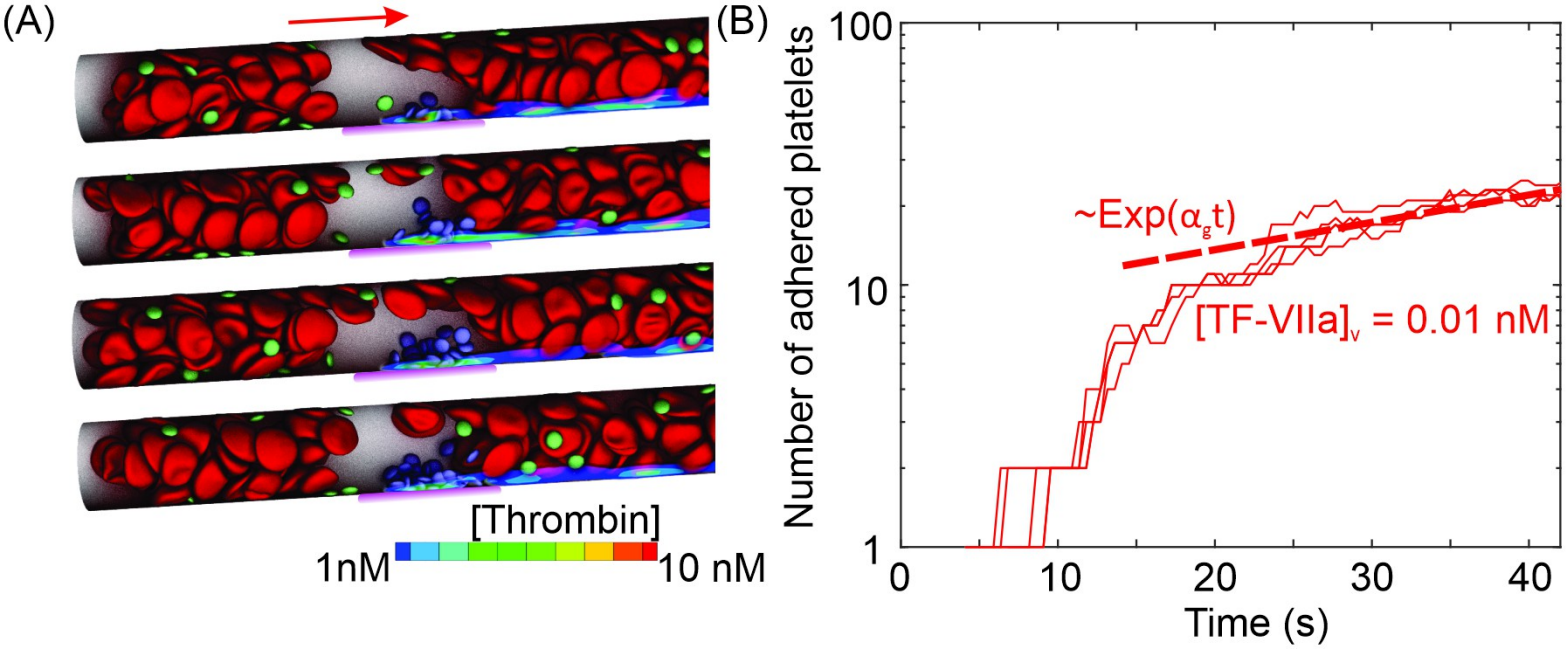
Platelet activation and adhesion driven by the coagulation cascade at the thrombogenic site. (A) Four sequential snapshots of platelets activation and adhesion at the thrombogenic site (highlighted by magenta). Passive platelets (green) become activated and adhesive after exposing to [FIIa] ≥ 1 nM, which is described by the concentration field in the figure. RBCs close to the thrombogenic site are not plotted for virtualization of the platelet aggregate. Blood flows from left to right. See S1 Movie. (B) Typical examples of the number of adhered platelets at the thrombogenic site vs. time (t), plotted in semi-log axes. The exponential growth rate is computed by fitting the data using dotted lines. Each of the growth rate measurement consists of four stochastic realizations to compute the mean and standard deviation.

First, we impose [TF-VIIa]_*v*_ = 0.01 nM at the thrombogenic site (highlighted by magenta in Fig 3A) to initiate the coagulation cascade, which mimics the inflammatory responses of endothelial cells. A sequence of snapshots of the platelet activation and adhesion in the microvessel at a blood flow velocity of 0.65 mm/s are displayed in Fig 3A, which show that after entering the boundary layer of [FIIa] ≥ 1nM, the passive platelets (green) become activated (blue) and gradually accumulate at the site of inflammation. The number of platelets adhered at the thrombogenic site is recorded for a period of 40 seconds, through which we can compute the thrombus growth rate. We compute the mean and standard deviation of the thrombus growth rate using four different stochastic realizations of our model. As illustrated in Fig 3B, the increased number of adhered platelets with respect to time at the mean blood flow velocity of 0.65 mm/s is plotted on a semi-log axes, which shows an initial transient followed by a steady exponential growth, a trend similar to the observation of an *in vivo* study using mouse model [76] as well as the measurements from a computational investigation of thrombus formation in a 2D channel [77].

**Fig 4:**
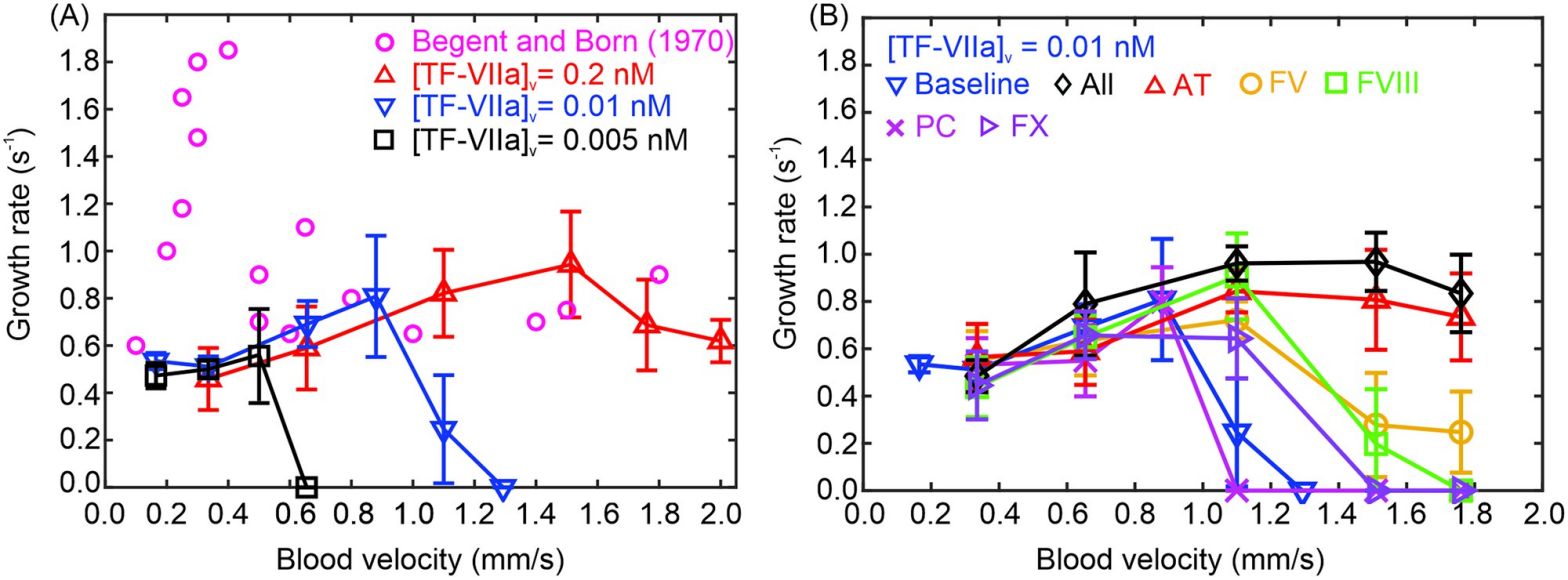
Impacts of inflammation levels, hemodynamics and aberrant levels of coagulation factors on the thrombus growth rate (*α*_*g*_). (A) Variation of thrombus growth rates with respect to different blood flow velocities at vascular expression of [TF-VIIa]_*v*_ = 0.005, 0.01 and 0.2 nM. (B) Variation of thrombus growth rates with respect to different blood flow velocities at aberrant levels of coagulation factors. Vascular expression of [TF-VIIa]_*v*_ is maintained at 0.01 nM for all cases. Guided by values listed in Table 1, [AT] is selected to be 45% of the reference value; [FVIII] is selected to be 470% of the reference value; [PC] is selected to be 156% of the reference value; [FV] is selected to be 181% of the reference value; [FX] is selected to be 125% of the reference value.

In the current work, we focus on the initiation and growing stages of microthrombi and thus we employ an exponential function to fit the relation between the number of adhered platelets and the simulation time in order to quantify the growing rates of microthrombi and also provide a means for determining how different biological variables affect the growth of microthrombi. This approach has been employed in former experimental [76] and computational studies [55, 78]. The thrombus growth rate is extracted by fitting the growth curve with exp(*α*_*g*_t), where *α*_*g*_ is the thrombus growth rate, as shown in Fig 3B. The thrombus growth rates computed at different flow velocities for [TF-VIIa]_*v*_ = 0.01 nM are summarized as the blue curve in Fig 4A. Our simulation results show that at lower blood velocities, thrombus grows relatively slow, likely caused by a low number of platelets transported to the thrombogenic site. As the blood velocity increases, more platelets are delivered to the thrombogenic site, contributing to the increase in the growth rate. The thrombus growth rate culminates at ∼0.78 s^−1^ at a blood velocity of 0.88 mm/s, after which it begins to decrease as velocity increases. The decreased growth rate at higher blood velocities is likely ascribed to the increased blood flow shear rate that inhibits the thrombus growth. In addition, strong blood flow tends to transport the generated FIIa downstream, leading to thinner FIIa boundary layers (see S1 Fig) and thus less platelet activation. Fig 4A shows that when the blood velocity is greater than 1.2 mm/s, no thrombus is formed because the strong advective effects of the blood flow reduce FIIa concentration at the thrombogenic site, leaving insufficient amount of FIIa to activate the platelets. Similar non-monotonic trends of thrombus growth rates varying with respect to blood flow velocities are observed in prior computational studies [77–79] as well as the experimental study by Begent and Born [76] (plotted in Fig 4A). The higher growth rates recorded from the experimental measurements are likely due to the usage of larger vessels (∼50 *μm*) as well as a different mechanism of triggering the thrombus formation (injecting ADP) [76].

Next, we vary [TF-VIIa]_*v*_ to 0.001 nM, 0.005 nM and 0.2 nM, respectively, to mimic different levels of inflammatory responses of endothelial cells and investigate how these variations affect the thrombus growth rate. Our results in Fig 4A show that at [TF-VIIa]_*v*_ = 0.005 nM, the non-monotonic relation between the thrombus growth rates and the blood velocities is maintained, but the maximum growth rate is decreased to 0.56 s^−1^ occurring at a blood velocity of 0.5 mm/s. No thrombosis occurs when the blood flow velocity is greater than 0.5 mm/s. When [TF-VIIa]_*v*_ is further reduced to 0.001 nM, FIIa generated at the thrombogenic site is insufficient to activate the platelets for all the examined flow velocities, resulting in no platelet activation and thrombus formation. On the other hand, when [TF-VIIa]_*v*_ is increased to 0.2 nM, the maximum growth rate increases to 0.94 s^−1^ at a blood velocity of 1.51 mm/s, above which the growth rate starts to decrease as the blood velocity increases. However, the decrease of growth rate is less pronounced compared to the cases of [TF-VIIa]_*v*_ = 0.005 nM and 0.01 nM. These results underline the role of hemodynamics in the coagulation-induced thrombosis and also imply that elevated inflammatory responses of endothelial cells could trigger thrombus formation not only under blood stasis but also under physiological flow conditions.

### COVID-19 induced hypercoagulability exacerbates thrombus formation

Our sensitivity analysis in the previous section suggests that reduced level of AT in the patients with COVID-19 play a key role in dictating FIIa generation in quiescent blood. In this section, we examine how the aberrant coagulation factors affect the thrombus formation in the flowing blood. Following the values listed in Table 1, we select the extreme values of abnormal coagulation factors measured from COVID-19 patients to perform our simulations. Specifically, AT is selected to be 45% of the reference value; FVIII is selected to be 470% of the reference value; PC is selected to be 156% of the reference value; FV is selected to be 181% of the reference value; FX is selected to be 125% of the reference value. [TF-VIIa]_*v*_ = 0.01 nM is selected to initiate the coagulation cascade at the thrombogenic site.

Our simulation results in Fig 4B show that similar non-monotonic relations between thrombus growth rates and blood velocities are observed for all the examined coagulation factors. When the blood flow velocity is less than 0.88 mm/s, the thrombus growth rates computed using the aberrant levels of coagulation factors are comparable to the growth rates obtained based on their reference values (blue curve). However, when the blood flow velocity is greater than 0.88 mm/s, the growth rates computed using the aberrant levels of AT, FV, FVIII and FX, become greater than the reference growth rate. Fig 4B shows that thrombus growth rates based on aberrant level of PC, FVIII and FX decrease to zero as the blood velocity is increased to 1.8 mm/s whereas notable thrombus growth is still observed in the case of FV and AT. These results suggest that the aberrant levels of coagulation factors could exacerbates thrombus formation by boosting FIIa generation to counteract the advective effect of blood flow on transporting FIIa downstream. We note that the impact of the aberrant level of AT on thrombus growth rate, which is similar to the overall impact of all the examined factors (black curve in Fig 4B), becomes more prominent as blood flow velocity increases. This finding indicates that in addition to dictating FIIa generation in the quiescent blood, AT functions as a key mediator for the thrombus growth in the flowing blood.

### White blood cells fuel the thrombus fire in COVID-19

Injured endothelial cells not only could initiate coagulation cascade by exposing TF, but also attract WBCs like neutrophils and monocytes through expressing cell adhesion molecules e.g. P-selectin [14, 80]. Activated and aggregated platelets at thrombogenic sites also can express a range of receptors, such as P-selectin and GP-Ib, that bind with rolling WBCs [15, 81]. The adhered WBCs were seen to activate coagulation cascade by expressing TF on cell surface even before platelet accumulation [82, 83]. Both the vascular and cellular expression of TF could contribute to the *in vivo* thrombus formation, but their relative contribution remains unclear [82]. Moreover, WBCs are involved in the formation of WBC-platelet clusters detected in the circulation of patients with COVID-19 [80, 84]. Clinical evidence shows that the levels of these WBC-platelet clusters in the patients under severe conditions are significantly higher than those with moderate disease [85], suggesting that these cell clusters may be implicated in the pathophysiology of COVID-19. In this section, we investigate the role of WBCs in initiating the microthrombus formation and explore the mechanism of WBC-platelet cluster formation.

**Fig 5:**
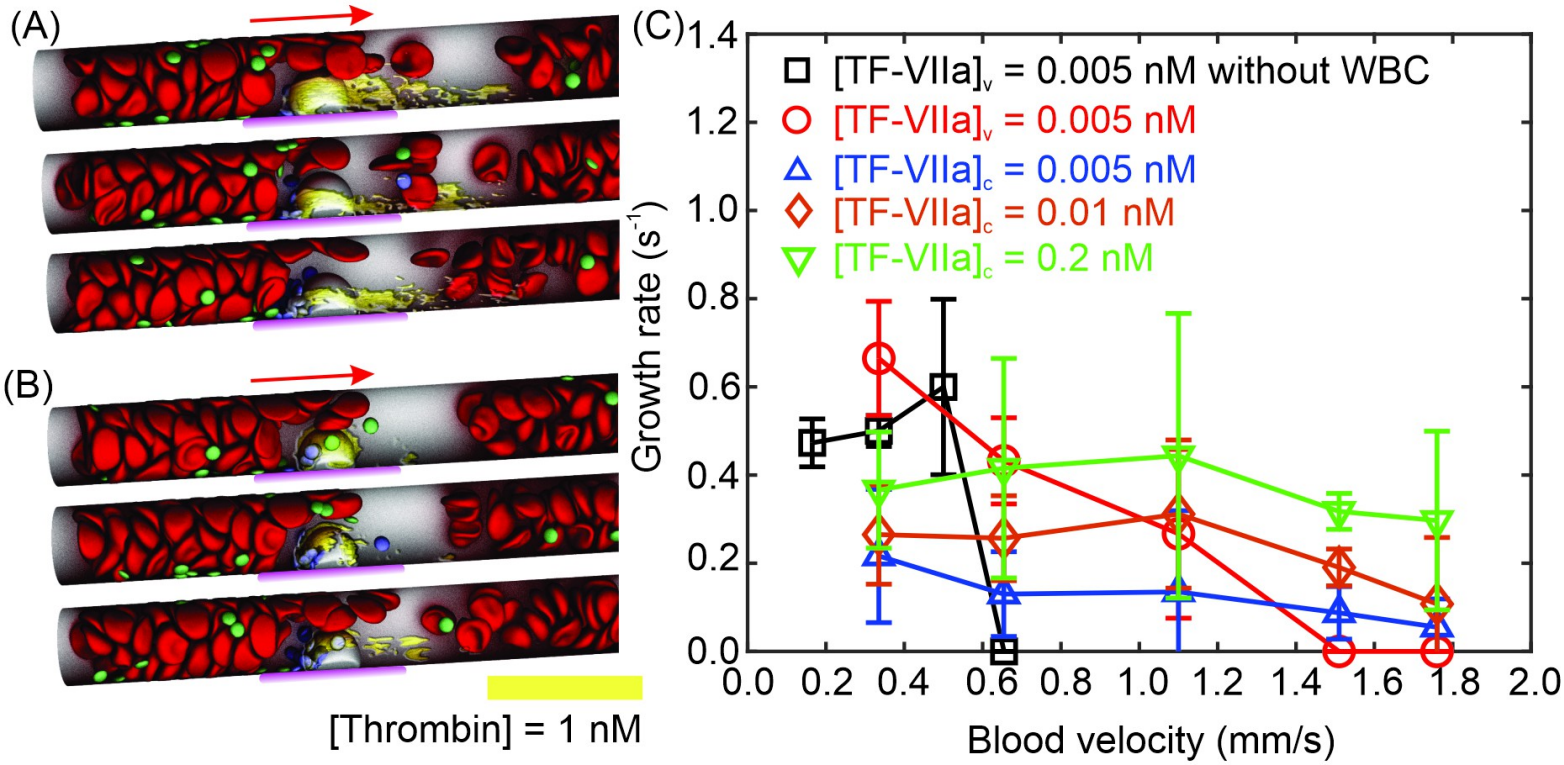
Adhesion of WBCs to the thrombogenic site precipitates the thrombus formation. Three sequential snapshots of platelet activation and adhesion at the thrombogenic site (highlighted by magenta) triggered by (A) vascular expression of [TF-VIIa]_*v*_ = 0.005 nM and (B) cellular expression of [TF-VIIa]_*c*_ = 0.005 nM on the adhered WBC. See S2 and S3 Movies. Passive platelets (green) become adhesive after exposing to [FIIa] ≥ 1 nM. Yellow color highlights the iso-surface of [FIIa] = 1 nM, within which the platelets become adhesive. RBCs close to the thrombogenic site are omitted for virtualization of the platelet aggregate. Blood flows at a velocity of 0.6 mm/s from left to right. (C) Variation of thrombus growth rates with respect to different blood flow velocities with and without presence of a WBC. [TF-VIIa]_*v*_ = 0.005 nM and [TF-VIIa]_*c*_ = 0.005 nM, 0.01 nM and 0.2 nM are examined, respectively.

First, we assume that a WBC is recruited firmly to a thrombogenic site before platelet accumulation, following the *in vivo* observation made by Darbousse et al. [82]. A boundary flux of [TF-VIIa]_*v*_ = 0.005 nM is employed to initiate the coagulation cascade from the thrombogenic site. Thrombus growth rates are evaluated at five different blood velocities varying from 0.33 mm/s to 1.76 mm/s. Fig 5A illustrates three sequential snapshots of platelet activation and aggregation at the thrombogenic site with the presence of a WBC at a blood velocity of 0.6 mm/s. We observe that a notable amount of FIIa (iso-surface of [FIIa] = 1 nM is highlighted as yellow) is generated upstream of the WBC, flowing around the WBC and eventually moving downstream (also see S2 Fig). Consequently, the passive platelets (green) become activated (blue) when approaching to the WBC and gradually accumulate at the headwind surface of the WBC. In contrast, when the WBC is not presented, our simulation results in Fig 5C(black curve) show that at [TF-VIIa]_*v*_ = 0.005 nM, no thrombus formation is observed at a blood velocity of 0.6 mm/s due to the insufficient amount of FIIa to activate platelets (see S2 Fig). This finding suggests that the presence of WBCs could promote the thrombus formation through intervening the transport of activated factors at the thrombogenic site. The thrombus growth rates computed with presence of a WBC at different blood velocities (red curve in Fig 5C) further indicate that recruitment of WBCs at the thrombogenic site could trigger thrombosis at higher blood velocities where no platelet activation and adhesion occurs before WBC recruitment (black curve). This finding demonstrates the biomechanical role of WBCs in exacerbating the pathological thrombus formation through altering the transport of coagulation factors.

Then, we examine the role of the adhered WBCs on initiating the coagulation cascade by expressing TF on the cell surface. We assume that a TF-positive WBC, which is firmly adhered at the thrombogenic site, initiates the coagulation cascade through cellular TF while the TF expression at the thrombogenic site is insufficient to activate the platelets, a condition corresponding to the mechanism of triggering arterial thrombosis by activated neutrophils in low-TF mouse models [82]. Similar to the initiation of coagulation cascade from vessel wall, we impose boundary fluxes of [TF-VIIa]_*c*_ = 0.005 nM, 0.01 nM and 0.2 nM on the cellular surface, respectively. FIIa boundary layer measured at a blood flow velocity of 0.6 mm/s in case of [TF-VIIa]_*c*_ = 0.005 nM is illustrated in Fig 5B (also see S3 Fig) which shows that FIIa is initially generated all over the surface of the WBC. Subsequently, the portion of FIIa at the tailwind surface of the WBC gradually fades away likely due to the lack of supply of inactivated coagulation factors. Eventually, FIIa generation only persists at the headwind surface of the WBC. As a result, Fig 5B shows that the passive platelets (green) become activated (blue) when flowing toward to the WBC and gradually accumulate at its headwind surface. Next, we compute the thrombus growth rates at five blood velocities varying from 0.33 mm/s to 1.76 mm/s for [TF-VIIa]_*c*_ = 0.005 nM, 0.01 nM and 0.2 nM, respectively. Our results in Fig 5C show that thrombus growth rates increase as the level of [TF-VIIa]_*c*_ is elevated, consistent with the trend of the thrombus development induced by the vascular expression of TF. However, thrombosis initiated from the cellular surface appears to be less sensitive to the varying blood flow velocities when compared to those initiated from the vascular wall, supporting the hypothesis that cellular expression of TF may play a key role in initiating thrombus formation at high blood flow rates followed by the involvement of the vascular TF for complementary thrombus growth [82].

### Interaction between WBCs and microthrombi contribute to the formation of circulating cell clusters in COVID-19

**Fig 6:**
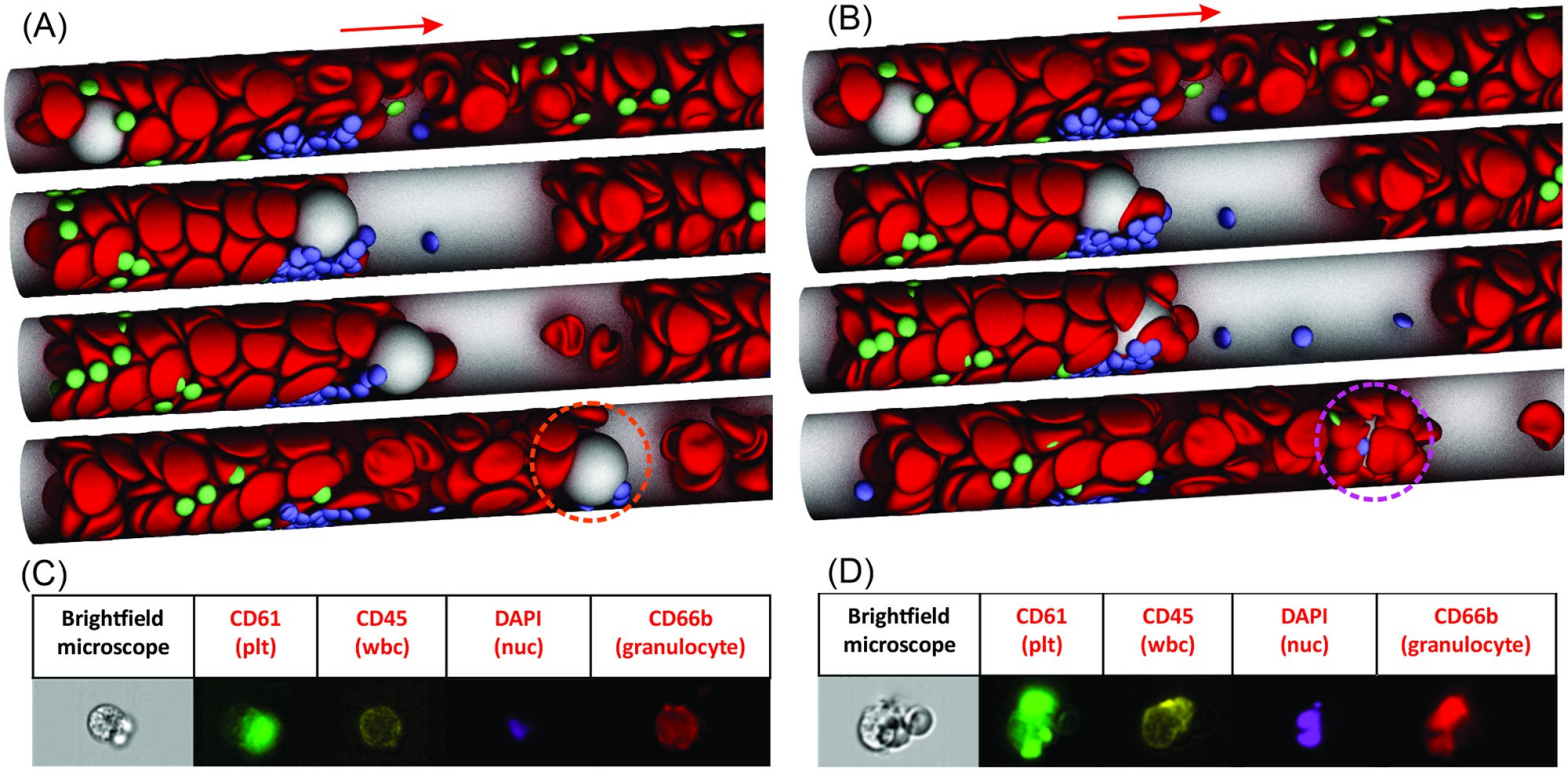
Interaction of a flowing WBC with existing thrombus. (A) Four sequential snapshots illustrate that at an initial blood flow velocity of 0.38 mm/s, a flowing WBC first attaches to an existing thrombus at the thrombogenic site and subsequently detaches from the thrombus, forming a WBC-platelet cluster (highlighted by an orange dotted circle) when WBC-RBC adhesion is not considered. See S4 Movie. (B) Under the same flow condition, the flowing WBC adheres to the surrounding platelets and RBCs during its interaction with the thrombus when WBC-RBC adhesion is considered, forming a WBC-RBC-platelet aggregate (highlighted by a magenta dotted circle). See S5 Movie. Blood flows from left to right. Flow cytometry images with staining of CD61, CD45, DAPI and CD66b show (C) a WBC-platelet cluster and (D) a WBC-platelet-RBC cluster from blood samples of patients with COVID-19 [84].

**Fig 7:**
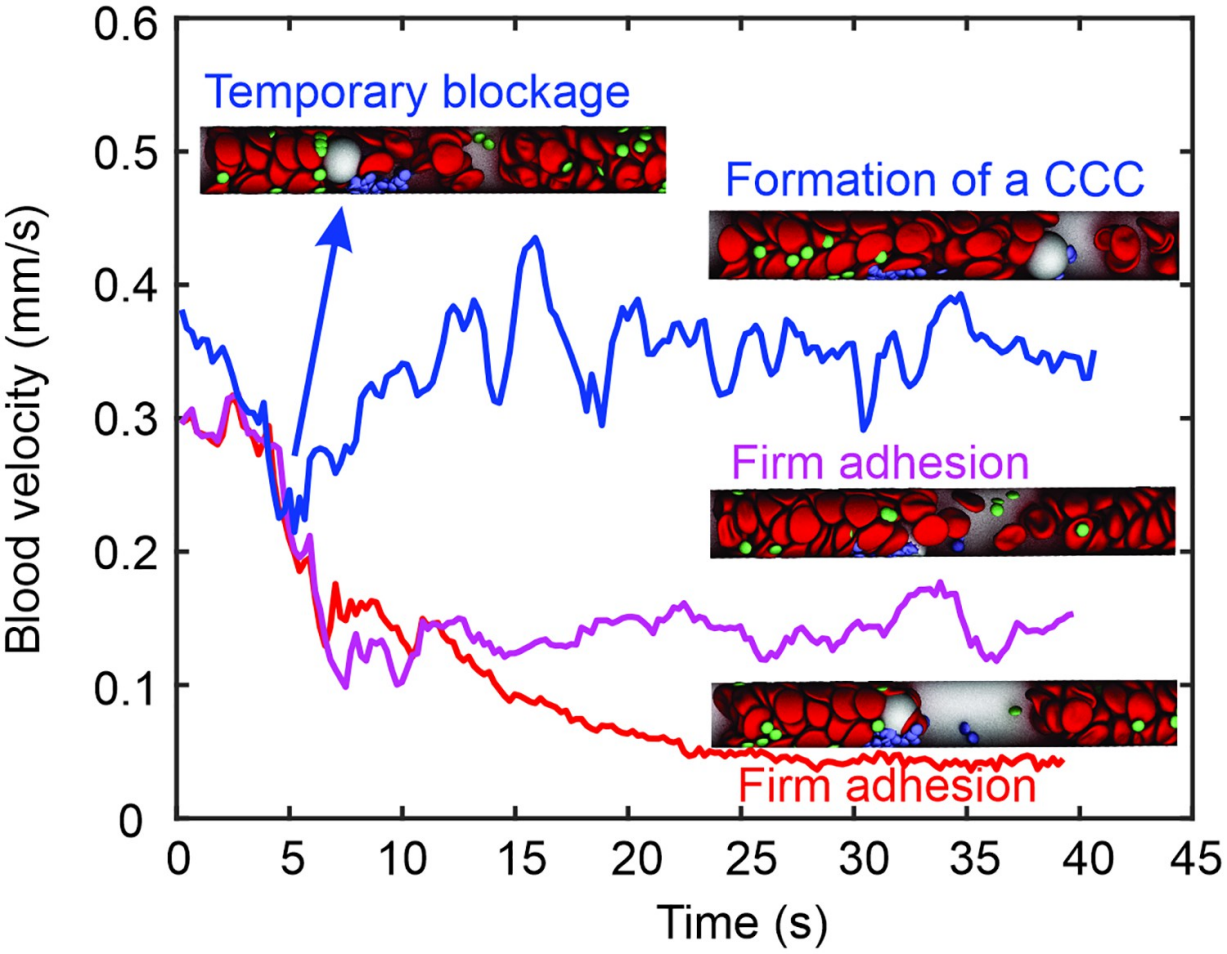
WBC-thrombus interaction affects the instantaneous mean blood velocity upstream of the thrombogenic site. Blue curve: when the mean blood velocity in the microvessel is 0.38 mm/s, the flowing WBC first attaches to the thrombus at the thrombogenic site and subsequently detaches from the thrombus. See S4 Movie. The instantaneous mean blood velocity upstream of the thrombogenic site first decreases induced by the short-term WBC-thrombus attachment and then recovers after the WBC detaches. Magenta curve: when the mean blood velocity is reduced to 0.3 mm/s, the WBC attaches to the thrombus at the thrombogenic site, establishing a firm adhesion. See S6 Movie. The instantaneous mean blood velocity upstream of the thrombogenic site decreases to ∼0.13 mm/s when the WBC-RBC adhesion is not considered. Red curve: under the same flow condition as the magenta curve, the instantaneous mean blood velocity upstream of the thrombogenic site further decreases to ∼0.05 mm/s when the WBC-RBC adhesion is considered. See S7 Movie.

Emerging clinical studies have shown that CCCs in the blood samples of patients with COVID-19 may have adverse impacts on physiological functions of the microvasculture, such as direct blockade of small blood vessels or serving as a nidus for new thrombus formation [80, 84–86]. However, the detailed mechanism causing the formation of these CCCs is elusive. In this section, we simulate a WBC rolling over an existing thrombus at the thrombogenic site, aiming to explore the underlying mechanism of CCC formation and investigate how the WBC-thrombus interaction affects the hemodynamics in the microvessels. The simulation setup is illustrated in Fig 6A where a WBC in the blood flow is moving toward a thrombus formed at the thrombogenic site. We vary the inlet blood flow velocities from 0.165 mm/s to 1.76 mm/s to examine its impact on the WBC-thrombus interaction. Our simulation results reveal that when the initial blood flow velocities are equal to or greater than 0.38 mm/s, which correspond to physiological flow conditions, the flowing WBC first attaches to the thrombus at the thrombogenic site and subsequently detaches from the thrombus, forming a flowing WBC-platelet cluster (see Fig 6A). The corresponding instantaneous mean blood velocity upstream of the thrombogenic site in Fig 7 shows a sudden decrease due to the short-term attachment of the WBC with the thrombus and then recovers after the WBC detaches. This simulation result provides a possible mechanism for the formation of WBC-platelet clusters in patients with COVID-19 (see Fig 6C) [80, 84]. When the initial blood flow velocities are equal to or smaller than 0.3 mm/s, corresponding to potential stasis conditions, Fig 7(magenta curve) shows that the WBC attaches to the thrombus and establishes a firm adhesion, partially blocking the blood flow in the microvessel. The partial blockage of the microvessel by the WBC-platelet aggregate causes a significant drop of the blood velocity to ∼0.13 mm/s. This finding suggests that reduced blood flow rate facilitates the firm adhesion of WBCs to the thrombogenic site, aggravating the blood stasis and thrombus formation.

We note that activated platelets may stimulate neutrophils to release neutrophil extracellular traps (NETs), one of the innate immune responses to capture and kill invading bacteria, fungi and viruses [87]. This highly negatively charged NETs can also trap flowing platelets and RBCs and thus potentiate thrombosis in COVID-19 [88, 89]. To represent the effect of NETs, we assume that the WBC becomes adhesive to RBCs in the blood flow after it interacts the thrombus. The adhesive force is defined by a Morse potential which is introduced in the Models and Methods section. Fig 6B shows four sequential snapshot of a flowing WBC is rolling over an existing thrombus at a blood flow velocity of 0.38 mm/s when the WBC-RBC adhesion is considered. While the process of the WBC interacting with the thrombus is similar to the case when WBC-RBC adhesion is not considered, the observed CCC is featured with RBCs covering the surface of the WBC. This result offers an attractive explanation for the observation of WBC-platelet-RBC clusters from patients with COVID-19 [84] (see Fig 6D). When the initial blood flow velocities are equal to or smaller than 0.3 mm/s, Fig 7(red curve) shows that the adhesion between the WBC and RBCs leads to a more pronounced decrease of the blood flow velocity from 0.3 mm/s to ∼0.05 mm/s. These results indicate WBCs not only could initiate the blood coagulation but also may directly participate in the microthrombus growth, a similar mechanism as they contribute to deep vein thrombosis [83]. In particular, trapping RBCs at the recruitment site by NETs can precipitate the blood velocity reduction, thereby promoting thrombus formation and increasing the risk of vaso-occlusion. This finding provides a mechanistic rationale for a clinical finding that neutrophil aggregation and NET formation could lead to rapid occlusion of pulmonary microvessels in COVID-19 [90].

## Discussion and summary

In this work, we employ a multiphysics and multiscale framework to quantify the contributions of multiple prothrombotic factors to the extent of microthrombosis in COVID-19. We find that thrombus growth rates vary with blood flow velocities in a non-monotonic way. An increasing blood flow velocity first promotes the thrombus formation through transporting more platelets to the thrombogenic site until thrombus growth culminates, after which it starts inhibiting the thrombus development because a strong blood flow tends to wash out the generated FIIa that activates the platelets. Our results also show that the critical blood flow velocities where the thrombus growth rate peaks increase with the elevated level of inflammatory responses of endothelial cells. The interplay between the blood flow rate and inflammation level in determining the thrombus development implies that accurate assessment of the hemodynamic conditions and inflammation levels of patients with COVID-19 can help to evaluate their risk for experiencing thrombotic events.

We perform analysis on derangement in the levels coagulation factors caused by COVID-19, and our results based on quiescent and flowing conditions both suggest that the formation of microthrombi is most sensitive to the reduced level of AT. This finding rationalizes usage of UFH and LMWH, which exert their anticoagulant effects by activating AT, for in-hospital anticoagulant treatments [91]. However, we note that the efficacy of heparin may be compromised on patients with COVID-19 who experience AT deficiency due to either increased consumption or reduced production [10]. Thus, an alternative anticoagulant for COVID-19 patients who are resistant to heparin may be required to achieve therapeutic anticoagulation levels. Our simulations of thrombosis under blood flow conditions (see Fig 4B) indicate that the aberrant level of FV is another prominent contributor in triggering thrombosis and thus could be potentially used as a new target for anticoagulant treatment, in agreement with other key studies examining abnormalities in coagulation factor levels in patients with COVID-19 [25, 92].

In addition to the three components of Virchow’s triad, we investigated the role of WBCs on initiating microthrombus formation. Our results show that WBCs are likely to contribute both biochemically and biomechanically to the microthrombus formation. The biochemical contributions may originate from expression of TF on cell surface, which could either be synthesized by WBCs in response to inflammatory agents [93] or are acquired by binding monocyte/platelet-derived microparticles in the blood flow [94]. The biomechanical contributions result from the fact that recruitment of WBCs augments the effect of vascular TF through hindering the advective effect of blood flow on removing FIIa from the thrombogenic site, thereby promoting platelet activation and aggregation. Our simulation results also reveal that thrombosis initiated from the expression of TF on WBCs is less sensitive to blood flow compared to those initiated from the vascular wall, suggesting that cellular expression of TF may be essential in initiating thrombus formation at high flow rates followed by the involvement of the vascular TF for complementary thrombus growth [82]. Moreover, the role of cellular expression of TF could become more pronounced in the propagation phase of the thrombus development because adhered WBCs can continue to bind with TF-expressed microparticles from the blood whereas the vascular TF exposure is diminished as the platelets gradually cover the thrombogenic site.

Our simulations on the interaction between microthrombi and flowing WBCs illustrate that WBCs can either be recruited by the existing thrombi, or detach from the thrombogenic sites, depending on the blood flow velocities. Strong blood flow causes the detachment of WBCs from the thrombus, forming flowing WBC-platelet clusters, a possible mechanism for the formation of WBC-platelet clusters in the circulation of patients with COVID-19 [3, 84]. It also implies that the number of WBC-platelet clusters might be associated with extent of thrombosis in the microvasculature, thereby offering a compelling explanation for positive correlation between the number of platelet-WBC aggregates and the severity of the disease [84, 85]. On the other hand, weak blood flow facilitates the recruitment of WBCs to the existing thrombus, leading to narrowing of the blood vessels and drop of the blood velocities upstream of the adhesion sites. These hemodynamic alterations precipitate thrombus formation and increase the risk of vaso-occlusion. Our simulations also illustrate the role of NETs in potentiating blood velocity reduction through capturing the flowing blood cells. Since the involvement of WBCs predisposes to thrombogenesis, institution of agents that are capable of inhibiting WBC adhesion to endothelial cells or activated platelets as well as reducing NETs release could constitute pathogenetically oriented strategies to delay or diminish the microthrombus development in COVID-19 [13].

There are several limitations related to our simulation results. We assume that only activated platelets can adhere to the thrombogenic sites in order to evaluate the impact of the coagulation cascade on the thrombus formation. However, prior studies have shown that the platelets could bind to the thrombogenic surface before activation [95]. As a result, our simulation results could underestimate the number of platelets adhered to the thrombogenic sites. In addition, we focus on the initial stage of thrombus formation and did not consider the aberrant fibrinolysis in COVID-19, e.g., fibrinolysis shutdown in critically ill patients, which also contributes to the increased incidence of thromboembolic events [96]. While we focus on the cellular expression of TF on WBCs in the current work, there is evidence that WBCs express other coagulation factors upon activation, such as FXa and FIIa, which may lead to the support of other anticoagulant strategies [13, 97]. We note that WBCs under proinflammatory state release proinflammatory molecules, such as granular enzymes and cytokines, which activate both intrinsic and extrinsic coagulation pathways [13]. In addition to WBCs, injured endothelial cells and activated platelets release cytokines that cause further endothelial cell dysfunction and inflammation, such as activation of the complement and kallikrein system [12]. Detailed simulation of these immune thrombotic processes requires more comprehensive kinetic models that combine aspects of coagulation with the complement system, which is not covered in the current study.

We note that oxygen saturation for patients with COVID-19 could drop from normal range of 95% to a level below 90% [98–100], which is one of the main causes of end-organ damage, such as acute kidney injury, liver injury, and pulmonary injury that leads to the need for mechanical ventilation. Based on multiple autopsy studies for patients who passed away from COVID-19-related sequelae [3–8], microthrombosis has been considered as one of the major causes of hypoxia both at the systemic level, such as in a low blood oxygen level due to microthrombi in the lungs, and at the local level of hypoxic injury to end-organs due to compromised blood perfusion that leads to kidney and/or liver injury. On the other hand, reduced oxygen saturation level can trigger activation of the coagulation system, but also down-regulate the physiologic anticoagulant pathways and endogenous fibrinolysis via hypoxia inducible factors (HIF) [101]. HIF can cause upregulated TF expression [102] and elevated level of inflammatory cytokines [103–105], such as tumor necrosis factor-*α* and Interleukin 6, which could also boost TF expression. In addition to upregulating the expression of TF, hypoxia could promote the thrombus formation through downregulating anticoagulants including protein S [106], a cofactor to protein C in the inactivation of FVa and FVIIIa, as well as tissue factor pathway inhibitor (TFPI) [107, 108], an anticoagulant protein that blocks the initiation of coagulation by inhibiting TF-VIIa. Furthermore, hypoxia contributes to the microvasculature thrombosis by suppressing the fibrinolytic process through boosting the activity of plasminogen activator inhibitor 1 (PAI-1) [109, 110], the principal inhibitor of fibrinolysis. Although many proinflammatory and procoagulant factors have been reported to be associated with hypoxia, there is a lack of models to quantify the connections among hypoxia, proinflammatory and procoagulant factors and the pathological thrombus formation. Thus, we could not consider the impact of hypoxia using the proposed framework. We hope that the current study can stimulate and steer new quantitative experimental and computational studies to explore the impact of oxygen saturation on the blood coagulation and thrombus formation, thereby providing inspirations to identify novel therapeutic targets to treat patients with COVID-19.

In summary, we employ a multiphysics computational framework to simulate the thrombus formation in a microvessel and quantify the connection between multiple prothrombotic factors in COVID-19 and the extent of microthrombus formation. The simulation results in the current study improve our understanding of the pathogenesis and pathophysiology of microthrombosis in COVID-19, identify the key factors that mediate the thrombus formation and provide insights to explore new therapeutic approaches for prophylaxis and treatment of COVID-19-associated thrombosis. A key feature of this employed framework is that both of its biophysical and biochemical components can be refined by future experimental data and further development of computational models to improve the capability and accuracy of the model predictions. Thus, it offers possibilities to explore and quantify the complex links between the inflammation and blood coagulation, thereby facilitating the discovery of new antithrombotic targets for treating other hyperinflammatory prothrombotic disorders.

## Supporting information

S1 Text Hydrodynamics and particle-based blood cell models. Table A. DPD parameters used in simulations. Table B. Cell membrane parameters for RBCs, platelets and WBCs. Table C. Morse potential parameters for cell-cell interactions.
(PDF)

S2 Text Platelet and WBC adhesion dynamics models. Table A. Values for bond formation and breakage kinetics for platelet-platelet and platelet-wall adhesion. Table B. Values for bond formation and breakage kinetics for leukocyte-platelet and leukocyte-wall adhesion.
(PDF)

S3 Text Coagulation factor transport and chemical kinetics model. Table A. List of factors in the coagulation cascade along with their initial concentrations. Table B. Reaction equations for source terms. Table C. Flux boundary conditions at the injured vessel wall region.
(PDF)

S1 Fig The impact of hemodynamics on the concentration boundary layers of thrombin ([IIa]). (TIF)

S2 Fig The impact of WBCs on the concentration boundary layers of thrombin ([IIa]). (TIF)

S3 Fig Four sequential snapshots of the development of concentration boundary layers of thrombin around a WBC measured at a blood velocity of 0.6 mm/s. (TIF)

S1 Movie Platelets activation and adhesion at the thrombogenic site at a blood velocity of 0.65 mm/s. The activation of platelets is triggered by vascular expression of [TF-VIIa]_*v*_ = 0.01 nM.
(MP4)

S2 Movie Platelets activation and adhesion at the thrombogenic site triggered by vascular expression of [TF-VIIa]_*v*_ = 0.005 nM when a WBC is adhered to the thrombogenic site. (MP4)

S3 Movie Platelets activation and adhesion at the thrombogenic site triggered by cellular expression of [TF-VIIa]_*c*_ = 0.005 nM on the adhered WBC. (MP4)

S4 Movie At a mean blood velocity of 0.38 mm/s, a flowing WBC first attaches to an existing thrombus at the thrombogenic site and subsequently detaches from the thrombus, forming a WBC-platelet cluster, when WBC-RBC adhesion is not considered. (MP4)

S5 Movie At a mean blood velocity of 0.38 mm/s, a flowing WBC adheres to the surrounding platelets and RBCs during its interaction with a thrombus, forming a WBC-RBC-platelet aggregate, when WBC-RBC adhesion is considered. (MP4)

S6 Movie At a mean blood velocity of 0.3 mm/s, a flowing WBC attaches to the thrombus at the thrombogenic site, establishing a firm adhesion, which causes a partial blockage of the blood vessel when WBC-RBC adhesion is not considered. (MP4)

S7 Movie At a mean blood velocity of 0.3 mm/s, a flowing WBC attaches to the thrombus at the thrombogenic site, establishing a firm adhesion, which causes a full blockage of the blood vessel when WBC-RBC adhesion is considered. (MP4)
