# Supplementary material for: Multiphysics and multiscale modeling of microthrombosis in COVID-19
Source: PLoS Comput Biol. 2022 Mar 7;18(3):e1009892. doi: 10.1371/journal.pcbi.1009892 (PMC8901059; doi:10.1371/journal.pcbi.1009892)
Supplement: S1 Text — Table A. DPD parameters used in simulations. Table B. Cell membrane parameters for RBCs, platelets and WBCs. Table C. Morse potential parameters for cell-cell interactions. (PDF) [file pcbi.1009892.s001.pdf]

Multiphysics and multiscale modeling of microthrombosis in COVID-19

He Li, Yixiang Deng, Zhen Li, Ander Dorken Gallastegi, Galit H. Frydman, Christos S. Mantzoros, George E. [illegible]

S1 Text. Hydrodynamics and particle-based blood cell models

In this work, we employ dissipative particle dynamics method (DPD) to model blood flow, including plasma, red blood cells (RBCs), platelets and white blood cells (WBCs, leukocytes). The DPD method is a mesoscopic particle-based simulation technique, where each DPD particle represents a lump of molecules and they interact with other particles through soft pairwise forces (1, 2). DPD can provide the correct hydrodynamic behavior of fluids at the mesoscale, and it has been successfully applied to study complex fluids (3–6). The equation of motion for each particle i is governed by the sum of pair interactions $\mathbf{f}_i$ with the surrounding particles j and it is integrated using a velocity-Verlet algorithm. The time evolution of velocity ($\mathbf{v}_i$) and position ($\mathbf{r}_i$) of a particle i with mass m_i is determined by Newton's second law of motion:

$$\mathrm{d}\mathbf{r}_i = \mathbf{v}_i \,\mathrm{dt}\,; \quad \mathrm{d}\mathbf{v}_i = \mathbf{f}_i/m_i \,\mathrm{dt}. \tag{1}$$

In DPD method, the total force $\mathbf{f}_i$ exerted on particle i by particle j is composed of a conservative force ($\mathbf{F}_{ij}^C$), a dissipative force ($\mathbf{F}_{ij}^D$), and a random force ($\mathbf{F}_{ij}^R$) given by

$$\mathbf{F}_{ij}^C = a_{ij}(1 - \frac{r_{ij}}{r_c})\hat{\mathbf{r}}_{ij} \quad for \quad r_{ij} \le r_c; \quad 0 \quad for \quad r_{ij} > r_c, \tag{2}$$

$$\mathbf{F}_{ij}^D = \gamma\omega_d(r_{ij})(\hat{\mathbf{r}}_{ij} \cdot \hat{\mathbf{v}}_{ij})\hat{\mathbf{r}}_{ij}, \tag{3}$$

$$\mathbf{F}_{ij}^R = \sigma\omega_r(r_{ij})\frac{\zeta_{ij}}{\sqrt{dt}}\hat{\mathbf{r}}_{ij}, \tag{4}$$

where r_c is a cut-off radius, and a_{ij}, γ, σ are the conservative, dissipative, random coefficients, respectively, r_{ij} is the distance between two particles with the corresponding unit vector $\hat{\mathbf{r}}_{ij}$, $\hat{\mathbf{v}}_{ij}$ is the difference between the two velocities, ζ_{ij} is a Gaussian random number with zero mean and unit variance, and dt is the simulation timestep size. It is selected to be 0.001 in the current work. The parameters γ and σ and the weight functions coupled through the fluctuation-dissipation theorem and they are calculated by $\omega_d = \omega_r^2$ and $\sigma^2 = 2\gamma k_B T$, where k_B is the Boltzmann constant and T is the temperature of the system. The weight function $\omega_r(r_{ij}) = (1 - r_{ij}/r_c)^k$ with $k = 1$ in the standard DPD method (7), whereas other values of k have been used to increase the fluid viscosity (8, 9). The DPD parameters used in Eqs (2)-(4) for all types of DPD particles are given in Table A.

In addition to blood plasma modeled by collections of free DPD particles, the membrane of suspending cells including RBCs, platelets and WBCs is constructed by a 2D triangulated network with N_v vertices (DPD particles). The vertices are connected by N_s elastic bonds to impose proper membrane mechanics. These DPD representations of RBCs, platelets and WBCs were extensively used and validated in the previous studies for both healthy and diseased cells (5, 6, 9–11). For a single cell, the free energy (V_{cell}) is given by

$$V_{cell} = V_s + V_b + V_{a+v}. \tag{5}$$

Table A: DPD parameters used in simulations. r_c is a cut-off radius, a_{ij} is the conservative coefficient, γ is the dissipative coefficient, and k is the weight function exponent. In all simulations, we set the particle mass $m = 1$, and the thermal energy $k_BT = 0.10$ in DPD units. Note that S: solvent (representing plasma), R: RBC, P: platelet, W: WBC.

type	r_c	a_{ij}	γ	k
S-S	1.58	5.0	20.0	0.20
S-R	1.5	0.0	45.0	0.20
S-P/S-W	1.5	0.0	10.0	0.20
R-R	1.0	10.0	10.0	0.20
R-P/R-W	1.0	10.0	10.0	0.20
P-P/P-W/W-W	1.0	10.0	10.0	0.20

The elastic energy V_s representing the elastic interactions of the cell membrane is defined by

$$V_s = \sum_{j \in 1...N_s} \left[\frac{k_B T l_m (3x_j^2 - 2x_j^3)}{4p(1-x_j)} + \frac{k_p}{l_j} \right], \tag{6}$$

where p is the persistence length, k_p is the spring constant, k_BT is the energy unit, l_j is the length of the spring j, l_m is the maximum spring extension, and $x_j = l_j/l_m$. p and k_p are computed by balancing the forces at equilibrium and from their relation to the macroscopic shear modulus, μ_s:

$$\mu_s = \frac{\sqrt{3}k_B T}{4pl_m x_0} \left(\frac{x_0}{2(1-x_0)^3} - \frac{1}{4(1-x_0)^2} + \frac{1}{4} \right) + \frac{3\sqrt{3}k_p}{4l_0^3}, \tag{7}$$

where l_0 is the equilibrium spring length and $x_0 = l_0/l_m$. The bending resistance V_b of the cell membrane is modeled by

$$V_b = \sum_{j \in 1...N_s} k_b \left[1 - cos(\theta_j - \theta_0) \right], \tag{8}$$

where k_b is the bending constant, and it is related to the macroscopic bending rigidity k_c with the expression $k_b = 2k_c/\sqrt{3}$, θ_j is the instantaneous angle between two adjacent triangles sharing the common edge j, and θ_0 is the spontaneous angle. In addition, the area and volume constraints V_{a+v} are imposed to mimic the area-preserving lipid bilayer and the incompressible interior fluid. The corresponding energy is given by

$$V_{a+v} = \sum_{j \in 1...N_t} \frac{k_d (A_j - A_0)^2}{2A_0} + \frac{k_a (A_{\text{cell}} - A_0^{\text{tot}})^2}{2A_0^{\text{tot}}} + \frac{k_v (V_{\text{cell}} - V_0^{\text{tot}})^2}{2V_0^{\text{tot}}}, \tag{9}$$

where N_t is the number of triangles in the membrane network, A_0 is the equilibrium value of a triangle area, and k_d, k_a and k_v are the local area, global area and volume constraint coefficients, respectively. The terms A_0^{tot} and V_0^{tot} are targeted cell area and volume.

We model RBCs with a biconcave shape using N_v=500 DPD particles. The shear modulus and bending rigidity of the RBC model are selected to be μ_0 = 4.73 μN/m and k_0 = 2.4×10^{-19} J, respectively. The cell surface area is selected to be A_0^{tot} = 132.9 μm^2, and cell volume V_0^{tot} = 92.5 μm^3, which give a surface to volume ratio of S/V = 1.44. All parameters used in our RBCs model are validated based on existing experimental data from single RBC mechanics to blood flow dynamics (5, 9–11).

Table B: Cell membrane parameters for RBCs, platelets (PLTs) and WBCs. N_v is the number of DPD particles on the membrane, l_m is the maximum bond extension, l_0 is the equilibrium bond length, k_b is the bending constant, μ_s is the shear modulus, A_0^{tot} and V_0^{tot} are the specified cell area and volume, respectively, $k_d + k_a$ is the combined area constraint coefficient, and k_v is the volume constraint coefficient.

cell	N_v	l_m/l_0	k_b	μ_s	A_0^{tot} (V_0^{tot})	$k_d + k_a$ (k_v)
RBC	500	1.8	6.025	100.0	132.87 (92.45)	5000 (5000)
PLT	48	1.8	602.5	10^4	19.63 (6.02)	5000 (10^4)
WBC	2498	1.8	48.2	10^4	314 (523)	5000 (10^4)

The platelet model, which consists of N_v = 48 DPD particles, is assumed to have a discoid cell shape with an aspect ratio of AR = 0.38. Based on our previous analysis on the patient-specific data (12), the surface area and volume of a platelet in passive state is selected to be A_0^{tot} = 19.627 μm^2 and V_0^{tot} = 6 μm^3, respectively. Since platelets are nearly rigid in their passive form, we choose shear modulus and bending rigidity sufficiently large (μ_p = 100 μ_0 and k_p = 100 k_0) to ensure its mechanical behavior as a more rigid cell.

A passive WBC model, comprised of N_v = 2498 DPD particles, is represented by a spherical cell with a diameter of 10 μm, following our previous work (5, 6). A WBC is less deformable compared to RBCs with the estimated shear modulus of 300–3000 μN/m (13) and bending stiffness of 1–2$\times 10^{-18}$ J (14). Guided by these studies, we model a WBC with μ_l = 100 μ_0 and k_l = 8 k_0 in the current work. The cell membrane parameters used in eqn (6)-(9) for RBC, platelet and WBC models are summarized in Table B.

Table C: Morse potential parameters for cell-cell interactions. D_e is the well depth of the potential, r_0 is the zero force distance, and β characterizes the interaction range. Note that R = RBC, P = platelet, W = WBC.

type	D_e	β	r_0
R-R	5.0	2.0	1.0
R-P/R-W	10.0	2.0	1.0
P-P/P-W/W-W	10.0	2.0	1.0

In order to prevent cell overlap we also employ a Morse potential between cell membrane particles in the form of

$$V_M(r) = D_e[e^{2\beta(r_0 - r)} - 2e^{\beta(r_0 - r)}]\,, \tag{10}$$

where r is the separation distance, r_0 is the zero force distance, D_e is the well depth of the potential, and β characterizes the interaction range. By properly setting the parameters, we can ensure sufficiently strong repulsive forces between cell membrane particles to prevent their overlap. We present the Morse potential parameters used for cell-cell interactions in Table C. Note that the cutoff distance of $r_{M,cut} = 1$ is used for all the Morse interactions when WBC-RBC adhesion is not considered. When WBC-RBC adhesion is considered, $r_{M,cut}$ is selected to be 1.2 between RBCs and WBCs and $r_{M,cut} = 1$ for other cell-cell interactions.

In order to relate the DPD parameters with the physical values, we need to first define length and time

scales. The RBC membrane shear modulus imposes the time scale for the DPD system, which follows

$$[t] = [L]\frac{\eta^P}{\eta^M}\frac{\mu_s^M}{\mu_s^P} \tag{11a}$$

$$[F] = [L]\eta^M\frac{\mu_s^P}{\mu_s^M} \tag{11b}$$

where μ_s is the RBC membrane shear modulus, μ is the plasma viscosity, and superscripts M and P denote the model (DPD) and physical units, respectively. The length scale is taken as $[L] = 1 \times 10^{-6}m$, whereas the time scale is evaluated by Eq. (8) $[t] = 2.27 \times 10^{-4}s$ (using membrane shear modulus of healthy RBCs $\mu_s^p = 4.73 \times 10^{-6}N/m$ and plasma viscosity $\eta^P = 1.2 \times 10^3 Pas$). Further, chemical concentrations are scaled by $[C_0] = 1.0nM[L]^3/n$ where n is the DPD particles density.

References

1. Groot RD, Warren PB. Dissipative particle dynamics: Bridging the gap between atomistic and mesoscopic simulation. The Journal of Chemical Physics. 1997;107(11):4423–4435.
2. Espanol P, Warren P. Statistical mechanics of dissipative particle dynamics. EPL (Europhysics Letters). 1995;30(4):191.
3. Fedosov DA, Pan W, Caswell B, Gompper G, Karniadakis GE. Predicting human blood viscosity in silico. Proceedings of the National Academy of Sciences. 2011;108(29):11772–11777.
4. Ye T, Phan-Thien N, Lim CT. Particle-based simulations of red blood cells –a review. J Biomech. 2016;49(11):2255–2266.
5. Lei H, Karniadakis GE. Probing vasoocclusion phenomena in sickle cell anemia via mesoscopic simulations. Proceedings of the National Academy of Sciences. 2013;110(28):11326–11330.
6. Chang H, Yazdani A, Li X, Douglas K, Mantzoros CS, Karniadakis GE. Quantifying platelet margination in diabetic blood flow. Biophysical Journal. 2018;115(7):1371–1382.
7. Espanol P, Warren P. Statistical mechanics of dissipative particle dynamics. Europhys Lett. 1995;30(4):191.
8. Fan X, Phan-Thien N, Chen S, Wu X, Yong Ng T. Simulating flow of DNA suspension using dissipative particle dynamics. Phys Fluids. 2006;18(6):063102.
9. Fedosov DA, Caswell B, Karniadakis GE. A multiscale red blood cell model with accurate mechanics, rheology, and dynamics. Biophysical Journal. 2010;98(10):2215–2225.
10. Pivkin IV, Karniadakis GE. Accurate coarse-grained modeling of red blood cells. Physical Review Letters. 2008;101(11):118105.
11. Yazdani A, Karniadakis GE. Sub-cellular modeling of platelet transport in blood flow through microchannels with constriction. Soft Matter. 2016;12(19):4339–4351.
12. Chang HY, Li X, Karniadakis GE. Modeling of biomechanics and biorheology of red blood cells in type 2 diabetes mellitus. Biophysical Journal. 2017;113(2):481–490.
13. Jadhav S, Eggleton CD, Konstantopoulos K. A 3-D computational model predicts that cell deformation affects selectin-mediated leukocyte rolling. Biophysical Journal. 2005;88(1):96–104.
14. Zhelev DV, Needham D, Hochmuth RM. Role of the membrane cortex in neutrophil deformation in small pipets. Biophysical Journal. 1994;67(2):696–705.
