# Supplementary material for: Multiphysics and multiscale modeling of microthrombosis in COVID-19
Source: PLoS Comput Biol. 2022 Mar 7;18(3):e1009892. doi: 10.1371/journal.pcbi.1009892 (PMC8901059; doi:10.1371/journal.pcbi.1009892)
Supplement: S2 Text — Table A. Values for bond formation and breakage kinetics for platelet-platelet and platelet-wall adhesion. Table B. Values for bond formation and breakage kinetics for leukocyte-platelet and leukocyte-wall adhesion. (PDF) [file pcbi.1009892.s002.pdf]

Multiphysics and multiscale modeling of microthrombosis in COVID-19

He Li, Yixiang Deng, Zhen Li, Ander Dorken Gallastegi, Galit H. Frydman, Christos S. Mantzoros, George E. K[illegible]

S2 Text. Platelet and WBC adhesion dynamics models

In this work, we consider the platelet adhesion triggered by the interaction between the platelet receptor GPIbα and A1 domains of immobilized von Willebrand factor (vWF) on the vessel wall. Assuming a harmonic GPIbα-vWF bond with stiffness k_b, the binding force F_b can be calculated as $F_b = -k_b(x_b - l_b)$, where x_b is the receptor-ligand distance and l_b is the equilibrium bond length. The values of the parameters in the platelet adhesive dynamics model are listed in Table A. Passive platelets can become activated by exposure to sufficiently high concentrations of thrombin, TxA2 and ADP. We define an activation function $\omega(x, t) = [\text{IIa}]/[\text{IIa}]_{thr} + [\text{ADP}]/[\text{ADP}]_{thr} + [\text{TxA2}]/[\text{TxA2}]_{thr}$, where the subscript 'thr' corresponds to the threshold concentration that activates the platelets. Platelets transporting through the regions with values of ω > 1 will become activated. In this study, we assume a negligible activating effect for TxA2, and the threshold values of $[\text{IIa}]_{thr} = 1nM$ (1) and $[\text{ADP}]_{thr} = 1000$ nM (2). Experimental measurements show that platelets release approximately 3×10^{-8} nM ADP to the blood stream within 5 seconds following activation (2, 3). Here, we assign a normal distribution for the release function $R(t) = \exp[-(t-\mu)^2/\sigma^2]/\sqrt{2\pi\sigma^2}$ with the mean release time of $\mu = 3$ s (seconds) and variance of $\sigma^2 = 2$ s^2 (second^2). We also assume that once the platelets become activated, they are ready to form bonds with the particles representing vWF ligands on the site of inflammation.

Table A: Values for bond formation and breakage kinetics for platelet-platelet and platelet-wall adhesion.

Parameter	Physical Value	Reference
k_f^0	40 s^{-1}	(4)
k_r^0	0.0022 s^{-1}	(4)
σ_f	1.8 nm	(4)
σ_r	1.62 nm	(4)
k_b	10 pN/nm	(5)
l_0	128 nm	(5)

We also employ a adhesive dynamics model to simulate the rolling and firmly adhesion of WBCs to the endothelial cells under inflammation, a part of the human immune response in COVID-19. Previous *in vivo* studies indicated that the binding between P-selectin expressed by the inflammation-activated vascular endothelium and the P-selectinglycoprotein- ligand-1 (PSGL-1) mediates the leukocyte rolling (6, 7). This finding has been confirmed by a number of computational studies (8–11). In addition, activated platelets may adhere to leukocytes, forming circulating mixed aggregates, a reliable marker that are associated with several cardiovascular conditions (12). The molecular mechanisms regulating this interaction also involve a central role of platelet P-selectin binding to PSGL-1 on leukocytes, which activates of the beta-2 integrin Mac-1,

leading to the firm adhesion between the two cell types. In this work, we employ the formulation developed by Dembo et al. (13) to compute the forward and reverse rates for the receptor-ligand bond between leukocytes and activated endothelium as well as between leukocytes and platelets. The model parameters are listed in Table B and these parameters have been used in the prior studies to model the rolling adhesion of leukocytes on endothelial cells (10, 11, 14). The firm adhesion between leukocytes and platelets as well as between leukocytes and thrombogenic sites is achieved by tuning the value of k_r^0 to mimic the effect of subsequent binding of other ligand-receptor pairs after the initial P-selectin-PSGL-1 binding, following the work of (10).

Table B: Values for bond formation and breakage kinetics for leukocyte-platelet and leukocyte-wall adhesion.

Parameter	Physical Value	Reference
k_f^0	30 s^{-1}	(14)
k_r^0	1.1 s^{-1}	(15, 16)
σ_{ts}	0.98 pN/nm	(13)
σ_b	1.0 pN/nm	(13)
l_0	100 nm	(14, 17)

References

1. Sorensen EN, Burgreen GW, Wagner WR, Antaki JF. Computational simulation of platelet deposition and activation: I. Model development and properties. Annals of Biomedical Engineering. 1999;27(4):436–448.

2. Hubbell JA, McIntire LV. Platelet active concentration profiles near growing thrombi. A mathematical consideration. Biophysical Journal. 1986;50(5):937–945.

3. Reed GL, Fitzgerald ML, Polgár J. Molecular mechanisms of platelet exocytosis: insights into the "secrete" life of thrombocytes. Blood, The Journal of the American Society of Hematology. 2000;96(10):3334–3342.

4. Kim J, Hudson NE, Springer TA. Force-induced on-rate switching and modulation by mutations in gain-of-function von Willebrand diseases. Proceedings of the National Academy of Sciences. 2015;112(15):4648–4653.

5. Mody NA, King MR. Platelet adhesive dynamics. Part II: high shear-induced transient aggregation via GPIbα-vWF-GPIbα bridging. Biophysical Journal. 2008;95(5):2556–2574.

6. Mayadas TN, Johnson RC, Rayburn H, Hynes RO, Wagner DD. Leukocyte rolling and extravasation are severely compromised in P selectin-deficient mice. Cell. 1993;74(3):541–554.

7. Dore M, Korthuis RJ, Granger DN, Entman ML, Smith CW. P-selectin mediates spontaneous leukocyte rolling in vivo. 1993;.

8. Hammer DA, Apte SM. Simulation of cell rolling and adhesion on surfaces in shear flow: general results and analysis of selectin-mediated neutrophil adhesion. Biophysical Journal. 1992;63(1):35–57.

9. Jadhav S, Eggleton CD, Konstantopoulos K. A 3-D computational model predicts that cell deformation affects selectin-mediated leukocyte rolling. Biophysical Journal. 2005;88(1):96–104.

10. Chang K, Tees DFJ, Hammer DA. The state diagram for cell adhesion under flow: leukocyte rolling and firm adhesion. Proceedings of the National Academy of Sciences. 2000;97(21):11262–11267.

11. Wu T, Qi D. Investigation of shear rates of rolling adhesion on leukocytes with bending of microvilli. Physical Review Fluids. 2019;4(6):063101.

12. Cerletti C, Tamburrelli C, Izzi B, Gianfagna F, De Gaetano G. Platelet-leukocyte interactions in thrombosis. Thrombosis Research. 2012;129(3):263–266.

13. Dembo M, Torney DC, Saxman K, Hammer D. The reaction-limited kinetics of membrane-to-surface adhesion and detachment. Proceedings of the Royal Society of London Series B Biological Sciences. 1988;234(1274):55–83.

14. Pappu V, Bagchi P. 3D computational modeling and simulation of leukocyte rolling adhesion and deformation. Computers in Biology and Medicine. 2008;38(6):738–753.

15. Mehta P, Cummings RD, McEver RP. Affinity and kinetic analysis of P-selectin binding to P-selectin glycoprotein ligand-1. Journal of Biological Chemistry. 1998;273(49):32506–32513.

16. Ramachandran V, Nollert MU, Qiu H, Liu W, Cummings RD, Zhu C, et al. Tyrosine replacement in P-selectin glycoprotein ligand-1 affects distinct kinetic and mechanical properties of bonds with P-and L-selectin. Proceedings of the National Academy of Sciences. 1999;96(24):13771–13776.

17. Marshall BT, Sarangapani KK, Wu J, Lawrence MB, McEver RP, Zhu C. Measuring molecular elasticity by atomic force microscope cantilever fluctuations. Biophysical Journal. 2006;90(2):681–692.
