# Supplementary material for: Multiphysics and multiscale modeling of microthrombosis in COVID-19
Source: PLoS Comput Biol. 2022 Mar 7;18(3):e1009892. doi: 10.1371/journal.pcbi.1009892 (PMC8901059; doi:10.1371/journal.pcbi.1009892)
Supplement: S3 Text — Table A. List of factors in the coagulation cascade along with their initial concentrations. Table B. Reaction equations for source terms. Table C. Flux boundary conditions at the injured vessel wall region. (PDF) [file pcbi.1009892.s003.pdf]

# Multiphysics and multiscale modeling of microthrombosis in COVID-19

He Li, Yixiang Deng, Zhen Li, Ander Dorken Gallastegi, Galit H. Frydman, Christos S. Mantzoros, George E. K

## S3 Text. Coagulation factor transport and chemical kinetics model

In the transport DPD (tDPD) model, the equations of concentration for each particle of mass $m_i$ are denoted by $dC_i = Q_i/m_i dt$ and integrated using a velocity-Verlet algorithm, where $C_i$ represents the concentration of species per unit mass carried by a particle, and $Q_i = \sum_{i \neq j}(Q_{ij}^D + Q_{ij}^R) + Q_i^S$ is the corresponding concentration flux. We note that $C_i$ can be a vector $C_i$ containing $N$ components, i.e., $C_1, C_2, ..., C_N$, when $N$ chemical species are considered. The total concentration flux accounts for the Fickian flux $Q_{ij}^D$ and random flux $Q_{ij}^R$, which are given by

$$Q_{ij}^D = -k_{ij}\omega_{DC}(r_{ij})(C_i - C_j), \quad (1)$$

$$Q_{ij}^R = \epsilon_{ij}\omega_{RC}(\xi_{ij})(\Delta t^{-1/2}), \quad (2)$$

where $k_{ij}$ and $\epsilon_{ij}$ determine the strength of the Fickian and random fluxes, and $Q_i^S$ represents the source term for the concentration due to chemical reactions. Further, the fluctuation-dissipation theorem is employed to relate the random terms to the dissipative terms, i.e., $\omega_{DC} = \omega_{RC}^2$, and the contribution of the random flux $Q_{ij}^R$ to the total flux is negligible (1). More detailed information about the tDPD model can be found in our previous work (1).

The ADR equations are in the form of

$$\frac{d[i]}{dt} = \Delta(D_i \Delta[i]) + Q_i^S \quad (3)$$

where $[i]$ denotes the concentration of $i^{\text{th}}$ factor and $D_i$ is the corresponding diffusion coefficient. Here, $Q_i^S$ is the nonlinear source term representing the production or consumption of $[i]$ due to the enzymatic reactions. See Table A for the list of the 23 biological factors, their diffusion coefficients and expressions to calculate the source terms for each factors. We note that the typical timescale of blood coagulation reactions is in the order of minutes and this makes the long-time tDPD computation very expensive. Therefore, following our previous work in (1), we keep the same Peclet number in our simulation to capture the correct flow physics, while we accelerate the reaction processes by 100 times to reduce the computational cost.

Here, we focus only on the coagulation cascade initiated by the tissue factor (TF) or extrinsic pathway to isolate and analyze the effect of TF-VIIa complex on the rate of thrombin production. Following the work of Anand et al. (2), the initial concentration of Fibrinogen (I), zymogens (II, V, VIII, IX, X, XI), inhibitors (ATIII, TFPI), and protein C (PC) are determined based on normal human physiological values which can be found the Table A, whereas concentrations of the enzymes (IIa, Va, VIIIa, IXa, Xa, XIa), fibrin and APC in plasma are initially zero or very small number. To initiate the coagulation, a spatially varied concentration level of subendothelium-bound TF-VIIa complex is prescribed at the site of inflammation, as shown in Fig.1(B) in the main text. This TF-VIIa complex drives four reactions at the wall (represented by flux conditions in Table C) that generate enzymes IXa and Xa which, in turn, initiate the extrinsic pathway.

Table A: List of factors in the coagulation cascade along with their initial concentrations $c_i^0$ and diffusion constants $D_i$ (2).

| Index | Species | Name | $c_i^0\ (nM)$ | $D_i \times 10^{-7}\ (cm^2/s)$ |
|---|---|---|---|---|
| 1 | IXa | enzyme IXa | $90 \times 10^{-5}$ | 6.25 |
| 2 | IX | zymogen IX | 90 | 5.63 |
| 3 | VIIIa | enzyme VIIIa | $0.7 \times 10^{-5}$ | 3.92 |
| 4 | VIII | zymogen VIII | 0.7 | 3.12 |
| 5 | Va | enzyme Va | $20 \times 10^{-5}$ | 3.82 |
| 6 | V | zymogen V | 20 | 3.12 |
| 7 | Xa | enzyme Xa | $170 \times 10^{-5}$ | 7.37 |
| 8 | X | zymogen X | 170 | 5.63 |
| 9 | IIa | thrombin | $1400 \times 10^{-5}$ | 6.47 |
| 10 | II | prothrombin | 1400 | 5.21 |
| 11 | Ia | fibrin | $7000 \times 10^{-5}$ | 2.47 |
| 12 | I | fibrinogen | 7000 | 3.1 |
| 13 | XIa | enzyme XIa | $30 \times 10^{-5}$ | 5.0 |
| 14 | XI | zymogen XI | 30 | 3.97 |
| 15 | ATIII | anti-thrombin-III | 2410 | 5.57 |
| 16 | TFPI | tissue factor pathway inhibitor | 2.5 | 6.30 |
| 17 | APC | active protein C | $60 \times 10^{-5}$ | 5.50 |
| 18 | PC | protein C | 60 | 5.44 |
| 19 | $\alpha_1$AT | $\alpha_1$-AntiTrypsin | 45000 | 5.82 |
| 20 | tPA | Tissue plasminogen activator | 0.08 | 5.28 |
| 21 | ADP | adenosine diphosphate | 0.0 | 25.70 |
| 22 | Z | tenase | $1.125 \times 10^{-4}$ | – |
| 23 | W | prothrombinase | 0.034 | – |

As mentioned in the main text, we use the coagulation model from Anand et al. (2). The list of factors and their normal initial concentration along with their diffusion coefficients in blood plasma are given in Table A. The equations governing the generation and depletion of the species ($S_i$) are formulated based on experimental data for the reaction kinetics, and are listed in Table B. The kinetic constants, also based on the experimental data, are given in the table's caption. Further, concentrations of two other chemical species tenase (Z) and prothrombinase (W) are computed through the relations [Z]=[VIIIa][IXa]/$K_{dZ}$ and [W]=[Va][Xa]/$K_{dW}$, respectively (2).

At the site of inflammation, we assume that the subendothelium-bound TF-VIIa complex drives the extrinsic pathway of the coagulation cascade through the subendothelium reactions that are represented by Neumann boundary conditions in the form of $-D_j\, \partial c_j / \partial n = Q_j$ . Surface reactions $Q_j$ along with their kinetic constants are given in Table C.

## References


1. Li Z, Yazdani A, Tartakovsky A, Karniadakis GE. Transport dissipative particle dynamics model for mesoscopic advection-diffusion-reaction problems. The Journal of Chemical Physics. 2015;143(1):014101.

2. Anand M, Rajagopal K, Rajagopal KR. A model for the formation, growth, and lysis of clots in quiescent

Table B: Reaction equations for source terms. The parameters are given as $k_9 = 2.54 \times 10^{-2}$, $K_{9M} = 160$, $h_9 = 3.74 \times 10^{-5}$, $k_8 = 0.449$, $K_{8M} = 1.12 \times 10^5$, $h_8 = 5.13 \times 10^{-4}$, $h_{C8} = 2.36 \times 10^{-2}$, $H_{C8M} = 14.6$, $k_5 = 6.24 \times 10^{-2}$, $K_{5M} = 140.5$, $h_5 = 3.93 \times 10^{-4}$, $h_{C5} = 2.36 \times 10^{-2}$, $H_{C5M} = 14.6$, $k_{10} = 5.523$, $K_{10M} = 160$, $h_{10} = 8.01 \times 10^{-4}$, $h_{TFPI} = 1.11 \times 10^{-3}$, $k_2 = 3.105$, $K_{2M} = 1060$, $h_2 = 1.65 \times 10^{-3}$, $k_1 = 8.177$, $K_{1M} = 3160$, $h_1 = 3.456$, $H_{1M} = 2.50 \times 10^5$, $k_{11} = 1.80 \times 10^{-5}$, $K_{11M} = 50$, $h_{11}^{A3} = 3.70 \times 10^{-6}$, $h_{11}^{L1} = 3.00 \times 10^{-8}$, $k_{PC} = 9.01 \times 10^{-2}$, $K_{PCM} = 3190$, $h_{PC} = 1.52 \times 10^{-9}$, $k_{PLA} = 2.77 \times 10^{-2}$, $K_{PLAM} = 18$, $h_{PLA} = 2.22 \times 10^{-4}$, $[ADP]_{plt} = 3 \times 10^{-8}\, nM$, $\mu = 3$ s, $\sigma^2 = 2$ s$^2$.

| |
|---|
| $S_1$ = ($k_9$[XIa][IX])/($K_{9M}$+[IX]) - $h_9$[IXa][ATIII] |
| $S_2$ = -($k_9$[XIa][IX])/($K_{9M}$+[IX]) |
| $S_3$ = ($k_8$[IIa][VIII])/($K_{8M}$+[VIII]) - $h_8$[VIIIa] - ($h_{C8}$[APC][VIIIa])/($H_{C8M}$+[VIIIa]) |
| $S_4$ = ($k_8$[IIa][VIII])/($K_{8M}$+[VIII]) |
| $S_5$ = ($k_5$[IIa][V])/($K_{5M}$+[V]) - $h_5$[Va] - ($h_{C5}$[APC][Va])/($H_{C5M}$+[Va]) |
| $S_6$ = -($k_5$[IIa][V])/($K_{5M}$+[V]) |
| $S_7$ = ($k_{10}$[Z][X])/($K_{10M}$+[X]) - $h_{10}$[Xa][ATIII] - $h_{TFPI}$[TFPI][Xa] |
| $S_8$ = -($k_{10}$[Z][X])/($K_{10M}$+[X]) |
| $S_9$ = ($k_2$[W][II])/($K_{2M}$+[II]) - $h_2$[IIa][ATIII] |
| $S_{10}$ = -($k_2$[W][II])/($K_{2M}$+[II]) |
| $S_{11}$ = ($k_1$[IIa][I])/($K_{1M}$+[I]) |
| $S_{12}$ = -($k_1$[IIa][I])/($K_{1M}$+[I]) |
| $S_{13}$ = ($k_{11}$[IIa][XI])/($K_{11M}$+[XI]) - $h_{11}^{A3}$[XIa][ATIII] - $h_{11}^{L1}$[XIa][$\alpha_1$AT] |
| $S_{14}$ = -($k_{11}$[IIa][XI])/($K_{11M}$+[XI]) |
| $S_{15}$ = -($h_9$[IXa]+$h_{10}$[Xa] + $h_2$[IIa] + $h_{11}^{A3}$[XIa])[ATIII] |
| $S_{16}$ = -$h_{TFPI}$[TFPI][Xa] |
| $S_{17}$ = ($k_{PC}$[IIa][PC])/($K_{PCM}$+[PC]) - $h_{PC}$[APC][$\alpha_1$AT] |
| $S_{18}$ = -($k_{PC}$[IIa][PC])/($K_{PCM}$+[PC]) |
| $S_{19}$ = -$h_{PC}$[APC][$\alpha_1$AT] - $h_{11}^{L1}$[XIa][$\alpha_1$AT] |
| $S_{20}$ = 0 |
| $S_{21}$ = [ADP]$_{plt}$ $\exp[-(t-\mu)^2/\sigma^2]/\sqrt{2\pi\sigma^2}$ |

plasma. A comparison between the effects of antithrombin III deficiency and protein C deficiency. Journal of Theoretical Biology. 2008;253(4):725–738.

Table C: Flux boundary conditions at the injured vessel wall region. The parameters are given as $k_{7,9} = 7.48 \times 10^{-2}$, $K_{7,9M} = 24$, $k_{7,10} = 0.238$, $K_{7,10M} = 240$.

| Index $j$ | Boundary flux terms $Q_j$ |
|---|---|
| 1 | $(k_{7,9}[2]^W[\text{TF-VIIa}]^W)/(K_{7,9M}+[2]^W)L$ |
| 2 | $-(k_{7,9}[2]^W[\text{TF-VIIa}]^W)/(K_{7,9M}+[2]^W)L$ |
| 7 | $(k_{7,10}[8]^W[\text{TF-VIIa}]^W)/(K_{7,10M}+[8]^W)L$ |
| 8 | $-(k_{7,10}[8]^W[\text{TF-VIIa}]^W)/(K_{7,10M}+[8]^W)L$ |
