# Supplementary figures and images for: Multiphysics and multiscale modeling of microthrombosis in COVID-19
Source: PLoS Comput Biol. 2022 Mar 7;18(3):e1009892. doi: 10.1371/journal.pcbi.1009892 (PMC8901059; doi:10.1371/journal.pcbi.1009892)

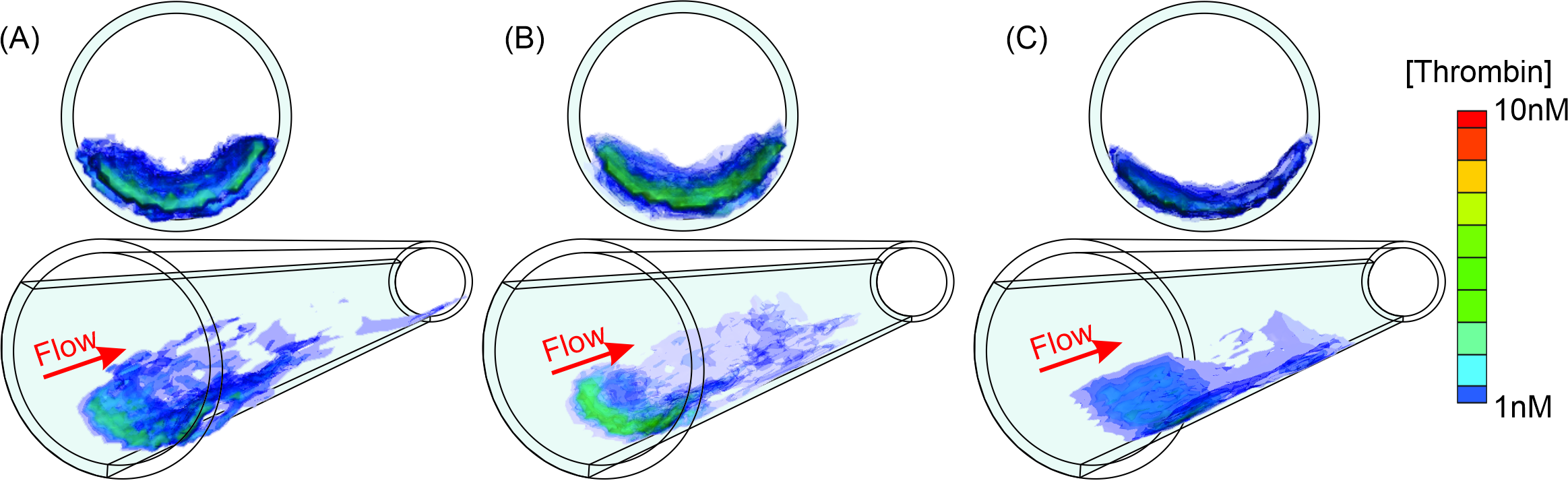

Supplement: S1 Fig — (TIF) [file pcbi.1009892.s004.tif]

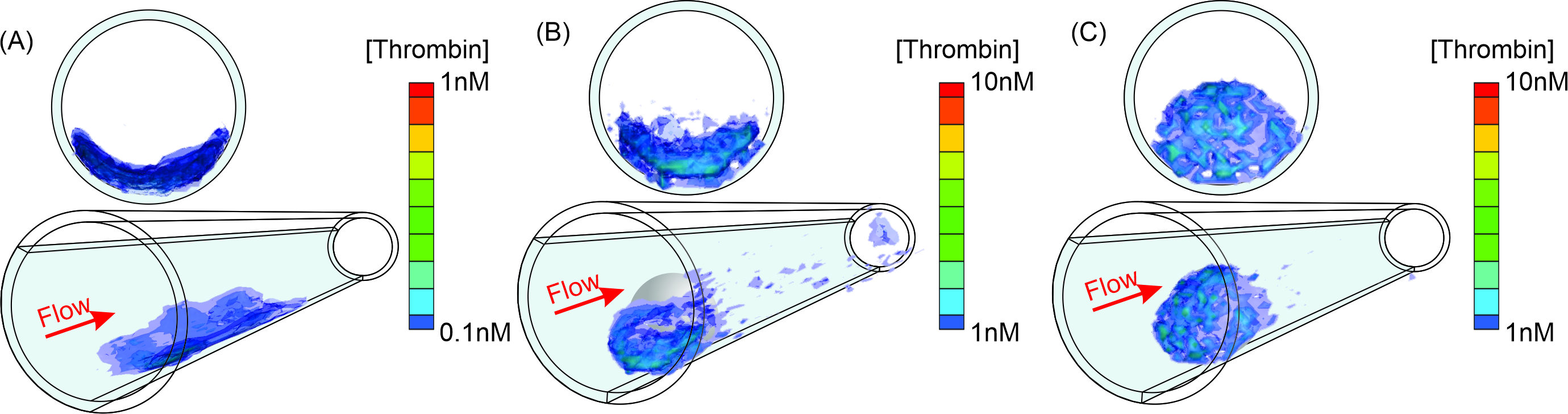

Supplement: S2 Fig — (TIF) [file pcbi.1009892.s005.tif]

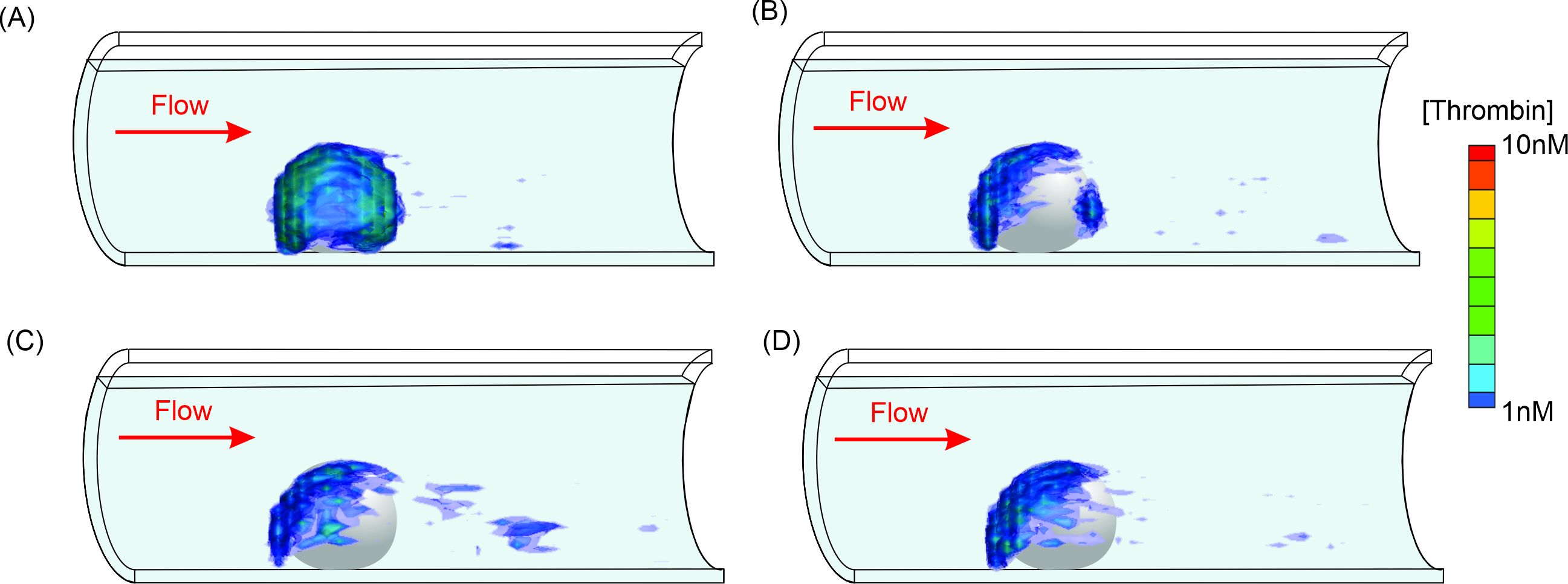

Supplement: S3 Fig — (TIF) [file pcbi.1009892.s006.tif]
